# Phenotype prediction in regulated metabolic networks

**DOI:** 10.1186/1752-0509-2-37

**Published:** 2008-04-25

**Authors:** Christoph Kaleta, Florian Centler, Pietro Speroni di Fenizio, Peter Dittrich

**Affiliations:** 1 Bio Systems Analysis Group, Department of Mathematics and Computer Science, Friedrich Schiller University Jena, Germany; 2 Jena Centre for Bioinformatics, Jena, Germany; 3 Research Institute in Networks & Communications Engineering (RINCE), Dublin City University, Ireland; 4 Helmholtz Centre for Environmental Research – UFZ, Department of Environmental Microbiology, Leipzig, Germany

## Abstract

**Background:**

Due to the growing amount of biological knowledge that is incorporated into metabolic network models, their analysis has become more and more challenging. Here, we examine the capabilities of the recently introduced chemical organization theory (OT) to ease this task. Considering only network stoichiometry, the theory allows the prediction of all potentially persistent species sets and therewith rigorously relates the structure of a network to its potential dynamics. By this, the phenotypes implied by a metabolic network can be predicted without the need for explicit knowledge of the detailed reaction kinetics.

**Results:**

We propose an approach to deal with regulation – and especially inhibitory interactions – in chemical organization theory. One advantage of this approach is that the metabolic network and its regulation are represented in an integrated way as one reaction network. To demonstrate the feasibility of this approach we examine a model by Covert and Palsson (J Biol Chem, 277(31), 2002) of the central metabolism of *E. coli *that incorporates the regulation of all involved genes. Our method correctly predicts the known growth phenotypes on 16 different substrates. Without specific assumptions, organization theory correctly predicts the lethality of knockout experiments in 101 out of 116 cases. Taking into account the same model specific assumptions as in the regulatory flux balance analysis (rFBA) by Covert and Palsson, the same performance is achieved (106 correctly predicted cases). Two model specific assumptions had to be considered: first, we have to assume that secreted molecules do not influence the regulatory system, and second, that metabolites with increasing concentrations indicate a lethal state.

**Conclusion:**

The introduced approach to model a metabolic network and its regulation in an integrated way as one reaction network makes organization analysis a universal technique to study the potential behavior of biological network models. Applying multiple methods like OT and rFBA is shown to be valuable to uncover critical assumptions and helps to improve model coherence.

## Background

The analysis of metabolic networks aims at the understanding of metabolic capabilities of organisms to adapt to, and to maintain growth under different external and internal conditions. Various tools exist today to analyze and predict behavior of organisms solely based on metabolic network structure.

Important results have been obtained by applying methods like flux balance analysis [1], modeling by differential equations [2], stochastic simulations [3], or elementary flux mode analysis [4]. While some of these methods concentrate on the network as a whole, others like elementary flux modes decompose it into smaller parts that form functional modules. Chemical organization theory [5] aims at the understanding of reaction networks from both sides. Its basic aim is to identify parts of the network, or more precisely, sets of molecular species, that are likely to coexist on a long time scale without any of the species vanishing or other species appearing anew. This not only encompasses steady states of the network as might be identified by elementary flux mode analysis (see Ref. [6] for the relation between elementary modes and organizations), but also conditions in which there are positive productions of metabolites. Therefore it can be seen as a method that defines a mapping from a reaction network consisting of reactions and metabolites to a set of potential phenotypes [7] of the network as specified by the set of organizations it contains. The theory of chemical organizations has previously been applied to a model of the central sugar metabolism of *E. coli *[8]. It was shown that organizations in the model coincided with known growth phenotypes of *E. coli *under different growth conditions. The growth on each of the carbon sources glucose, lactose, and glycerol could be matched to a specific organization. However, as negative regulation for some genes was ignored because the down-regulation of genes cannot push gene expression levels below the basal level, some organizations represented biologically infeasible system states, for example the simultaneous uptake of all carbon sources.

In this paper, we present an approach to explicitly consider inhibitory regulatory interactions within the analysis of chemical organizations. This allows the more faithful and more precise consideration of biological network models, making the identification of all potential phenotypes of regulated metabolic networks possible. First, the basic concepts of the theory of chemical organizations are presented in the next section. Then, the approach to deal with inhibitory interactions is introduced. The method is then first applied to a subnetwork of a network model of the central metabolism of *E. coli *to predict growth phenotypes. Finally, the method is applied to the complete network model to predict the lethality of gene knockouts.

### Theory of Chemical Organizations

The theory of chemical organizations [5] provides a new method to analyze complex reaction networks. Extending ideas by Fontana and Buss [9], one main objective is to determine combinations of network species that are more likely to be present over long periods of (simulation-) time than others. Such sets of species are called organizations. To be an organization, a species set has to fulfill two properties: algebraic closure and self-maintenance. The first property – closure – ensures that given the molecular species of an organization, there is no reaction within the reaction network that could create a novel species not yet present in the organization. The second property – self-maintenance – guarantees that every molecular species that is consumed within the organization can be recreated from organization species at a suffcient rate for its maintenance. The basic concepts required for this paper are summarized now more formally.

#### Reaction network

Let M be a set of molecular species, $P_{M}(M)$ denotes the set of all multisets with elements from M. A multiset differs from a set in the fact that it can contain the same element more than once. The set of reactions R occurring among the species M can then be defined by a relation $R\subseteq P_{M}(M)\times P_{M}(M)$. We call the pair 〈M,R〉 a *reaction network*.

#### Closed set

A set of species S⊆M is *closed*, if for all reactions (A→ℬ)∈R with $A\in P_{M}(S)$, then also $ℬ\in P_{M}(S)$. In other words: if the educts of a reaction are contained in S, then also its products must be in S. There is no reaction in R that could create any new species not yet in S from species contained in S.

#### Self-maintaining set

Given a reaction system 〈M,R〉 with *m *= |M| species and *n *= |R| reactions, **S **= (*m*_*i*, *j*_) be its *m *× *n *stoichiometric matrix, where *m*_*i*, *j *_is the number of molecules of species *i *that is produced in reaction *j *(*i.e*., right hand side minus left hand side). A set of species S⊆M is called *self-maintaining *if a flux vector *v *= (*v*_1_, *v*_2_, ..., *v*_*n*_) ∈ ${\mathbb{R}}_{\geq 0}^{n}$ exists such that the following three conditions are fulfilled:

(1) For every reaction (A→ℬ)∈R with $A\in P_{M}(S)$, its corresponding flux is $v_{A\to ℬ}>0$.

(2) For every reaction (A→ℬ)∈R with $A\notin P_{M}(S)$, its corresponding flux is $v_{A\to ℬ}=0$.

(3) For every species *i *∈ S, its concentration change is nonnegative: (**S***v*)_*i *_≥ 0.

In other words: if we consider only the sub-network made up by the species of S and additionally the species that can be created from S (but are not in S) (conditions (1) and (2)), we can find a positive flux vector, such that no species of S decays (condition (3)).

#### Organization

A set of species S⊆M that is closed and self-maintaining is called an *organization*.

#### Balanced organization

An organization S⊆M is a *balanced organization*, if a flux vector conforming to the self-maintenance condition can still be found, if in requirement 3 of the self-maintenance definition, the greater equal condition is replaced by equality:

(3') For every species *i *∈ S, its concentration change is zero: (**S***v*)_*i *_= 0.

A rigorous link between organizations and the potential dynamics of a reaction system is provided by Theorem 1 from Ref. [5]: Assume that the dynamics is modeled as a "chemical" differential equation system *dx*(*t*)*/dt *= **S***v*(*x*(*t*)), then all steady states of the system are instances of organizations. In other words, the species with concentration levels greater than zero in a particular steady state are exactly those species contained in a corresponding organization. Note that organizations do not necessarily contain a steady state, as they can also embody growth in which species have increasing concentrations. The only assumption made for this theorem is that molecules that are present and can react will react sooner or later (formally: $v_{A\to ℬ}(x)>0$, if and only if for all *i *∈ A: *x*_*i *_> 0). Note that this assumption differs fundamentally from the assumption made by methods like elementary mode analysis, which assume that each reaction can be switched off independently even if the reactants are present in large concentrations [6].

### Computing Organizations

To compute organizations, the convex polyhedral cone P can be used which is defined by the *n *+ *m *inequalities *v *≥ **0 **and **S **· *v *≥ **0 **in the flux space ${\mathbb{R}}_{\geq 0}^{n}$. This cone contains all self-maintenance flux vectors as described in the self-maintenance definition. In order to find species sets that are self-maintaining and closed, the extreme rays spanning P are combined in a recursive fashion and the resulting species sets tested. The Supplement contains a detailed description of the algorithm, and outlines a heuristic approach to compute organizations for large network models, for which the runtime of the algorithm exceeds practical limits (see Additional file 1).

## Methods

Regulation has not yet been explicitly considered in the analysis using the theory of chemical organizations. The aim of this section is to elaborate a concept that allows us to also account for regulation. This concept makes it possible to also study regulated metabolic networks using organization theory. The basic idea is to map regulatory rules to normal chemical reaction rules. For inhibitory rules and rules using the direction of a reversible flux we introduce pseudo species representing the absence of a species and the direction of a flux, respectively.

### Types of Regulation

To examine the effects of regulation on chemical organizations we first need to discuss the general types of regulatory interactions that occur in biological systems in more detail.

Regulation appears on different levels in the cell, being carried out by a variety of biological entities (*e.g*., small molecules, proteins, RNA) acting on further biological target entities. As we are considered with reaction networks, we focus here on the regulation of reactions. Two different types of regulation have to be considered. The first type of regulation only changes the flux of the regulated reaction slightly. Certain types of autoregulation fall into this category. This kind of regulation does not change the structure of the reaction network and hence does not affect its organizational hierarchy. The second type of regulation is more drastic: it turns a reaction completely off or enables a formerly unavailable reaction. This is the case, for example, when the expression of a protein that catalyzes a reaction is suddenly repressed. As a consequence, the catalyzed reaction is not available to the network anymore once the protein is completely degraded. The induction of uptake pathways is an example for enabling novel reactions. New reaction pathways become suddenly available.

Note that, even though drastic, this kind of categorization of regulation leads to meaningful models, for example when translated into a boolean regulatory network [10] and also generalizes to discretization using more than two levels as used in Ref. [11].

Regulatory interactions do not happen instantly. The time delay between the onset of a regulatory event and its measurable effect in the system can vary between milliseconds (*e.g*., phosphorylation of proteins in signal cascades [12]) and minutes (*e.g*., changes in gene expression [13]). However, as we are here interested in the long term behavior of the system, we do not take different time scales of regulation into account.

### Modeling Regulatoy Interactions

Several approaches exist to represent and model regulatory interactions [14], for example, boolean logic [10,15,16], stochastic models [17], and differential equations [18]. Whereas some approaches require a very detailed knowledge of the mechanisms and kinetics behind the regulation, the representation of regulatory networks by boolean logic can be useful if the knowledge about the underlying kinetics is limited [11]. In this approach, the state *on *or *off *is assigned to a regulated reaction [15]. We will adopt this notion to model regulatory interactions.

Two types of regulatory events have to be considered: *activation*, in which a species is required in order to perform a certain reaction, and *inhibition*, in which a species inhibits a certain reaction and makes it unavailable. To model this kind of regulation we make use of the properties of reactions being similar to rewriting rules, where the left hand side is being replaced by the right hand side. Taking this approach, the molecules on the left hand side need to be present for the reaction to proceed. Additionally, regulatory events can be triggered not only by the presence or absence of a species, but also by a species being available in excess or not.

#### Activation

Activation or turning on a reaction by a specific species can be simply modelled by considering this species as a kind of a catalyst. In terms of rewriting rules this approach can be considered as an additional constraint on the presence of certain species for the reaction to proceed. By this, the reaction can only take place when the activating species is present. Being a catalyst, the activating species is not consumed within the reaction it catalyzes.

Let us consider the general case in which species *E *activates a reaction that transforms a reactant *A *into product *B*. In the absence of *E*, the reaction shall have a zero flux, while the flux shall become positive in the presence of *E *and *A*. A reaction *A *→ *B *activated by *E *becomes:

*E *+ *A *→ *E *+ *B*.

Note that adding *E *as a catalyst on the reactant and product side of the reaction equation does not change the stoichiometric matrix **S**. Still, one unit of *A *is being consumed to produce one unit of *B*. Therefore any flux vector that guarantees self-maintenance for a set of metabolites including *E *but without considering *E *as an activator, will also guarantee self-maintenance when *E *is added as a catalyst to model activation.

#### Inhibition

Handling inhibition is more diffcult. If inhibitor *I *inhibits a reaction, we could add an if-statement to each reaction that guarantees that the reaction is only available when *I *is absent. But since we intend to model regulation within the language of reactions, such if-statements would not fulfill our requirements. An alternative way to model inhibition is to understand it as another type of activation, that is, as the activation of a reaction by the absence of an inhibitor. For achieving this we have to introduce a pseudo species *Ī *that represents the absence of inhibitor *I*. In terms of rewriting rules, such species is just a constraint on the presence of a certain species for a reaction to take place. A reaction *A *→ *B *inhibited by *I *becomes:

*Ī *+ *A *→ *Ī *+ *B*.

Only in the absence of *I*, represented by pseudo species *Ī*, educt *A *can react to form product *B*.

#### Modelling flux-direction dependent regulation

Sometimes it can make sense to define regulatory rules that depend on the *direction *at which one or more reversible reactions operate. Two examples can be found in Covert and Palsson [19], the rule for the pyruvate response regulator (gene *pdhR*) and the rule for the catabolite activator protein (gene *cra*). Given a reversible reaction *r*: *A *↔ *B *and the following flux-direction dependent regulatory rule:

"If the flux of reaction *r *is positive (forward), then activate protein E.",

we map this regulatory rule to the following conventional reaction rules by introducing a pseudo species *f*_*r *_and its inverse counterpart $\bar{f_{r}}$:

(1) *r*_*f *_: *A *+ *f*_*r *_→ *B *+ *f*_*r*_

(2) $$r_{b}:B+\bar{f_{r}}\to A+\bar{f_{r}}$$

(3) *r*_*E *_: *A *+ *f*_*r *_→ *A *+ *f*_*r *_+ *E *

Because in consistent organizations (see below) either *f*_*r *_or $\bar{f_{r}}$ is present (but not both), the reaction rules assure that either the forward *r*_*f *_or the backward reaction rule *r*_*b *_can be active. Here, in Eq. (3), we use the presence of the molecular species *A *and *f*_*r *_as an indicator for the activity of reaction *r*_*f *_. Conversely, we can use the presence of the molecular species *B *and $\bar{f_{r}}$ as an indicator for the activity of the backward reaction *r*_*b*_.

If necessary, we can add a pseudo species like *f*_*r *_to every reversible reaction rule *r *in order to obtain an indicator for the direction in which it operates. Then, these indicators can even be combined to represent a more complex rule. Note that in Covert and Palsson [19], different flux-directions are logically combined to indicate a surplus of a molecular species (*e.g*., *PYR *for the regulation of *pdhR*; and *FDP *or *F6P *for the regulation of *cra*).

#### Consistent organization

Introducing pseudo species causes a problem, as now two network species represent the same molecular entity. When computing the organizations of such a network, some organizations might exist that contain both or neither of the two species. In both cases, the presence of the species is not clearly defined. Either the presence and absence is indicated simultaneously, or no statement is made at all. Consequently, we only consider those organizations in the remaining of this paper, in which for all species it is clearly defined whether they are present or not. We call such organizations consistent.

#### Consistent organization

An organization O⊆M is called a *consistent organization*, if for all species *S *∈ M for which there exists a pseudo species $\bar{S}\in M$ that indicates its absence, either *S *or $\bar{S}$ is contained in the organization.

In passing we note that this approach allows one to model more than two states of a molecule, for example different phosphorylation states.

### Modeling Boolean Logic

There are few cases where a reaction is regulated by a single molecule alone. In most cases regulation is more complex, for example if the availability of a reaction is being determined by the interaction of a set of proteins. In such cases we need to model regulation by a set of boolean functions. This section presents an approach to account for such functions on the level of regulation (see also [20]).

All binary boolean functions can be reduced to either *AND *or *OR*, and the negation *NOT*. The construction of a negation has been outlined above. In principle, it would be suffcient to present a method to implement *AND *or *OR*. However, we present methods for both to ease the process of converting regulation logics to chemical reactions.

First we consider the *AND *function. A typical regulation incorporating an *AND *function is the required presence of two activators to perform a reaction. If we consider activator *E*1 and activator *E*2 to be necessary for a reaction converting educt *A *into product *B *we can write:

*E*1 + *E*2 + *A *→ *E*1 + *E*2 + *B*.

Next, the *OR *function is considered. An example for this case is a reaction transforming educt *A *into product *B *that can alternatively be activated by two activators *E*1 and *E*2. The presence of one of these activators is suffcient to perform the reaction. In this case, the reaction is split into two parts; one that accounts for the presence of activator *E*1, and one that accounts for the presence of activator *E*2:

*E*1 + *A *→ *E*1 + *B*

*E*2 + *A *→ *E*2 + *B*.

Taking these two basic functions, it is possible to model all regulatory relationships that can be represented by boolean rules in metabolic networks [20].

### Example: Regulatory switch

**Figure 1:**
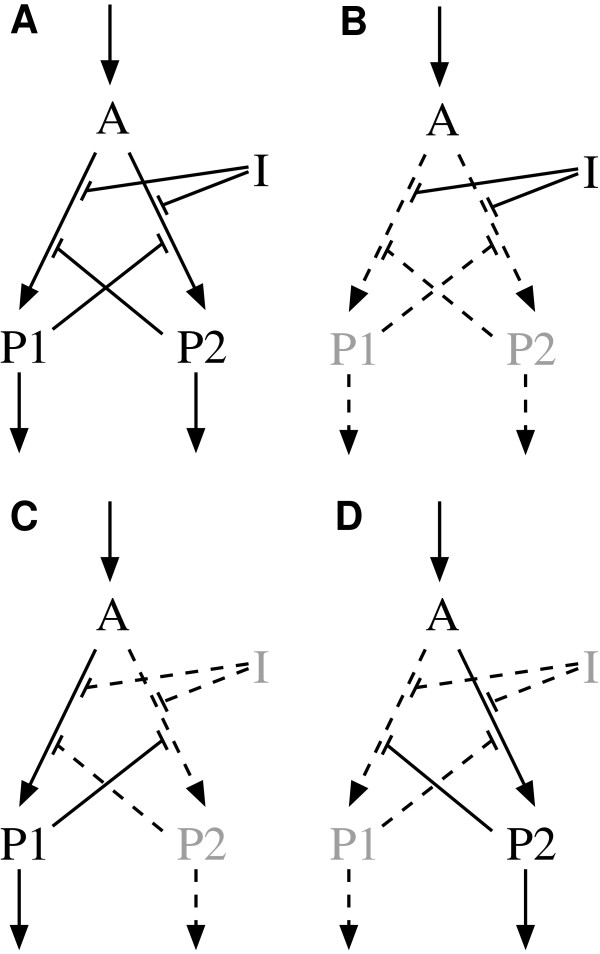
**A simple switch**. Regulatory switch network (**A**) and the reaction networks belonging to its three consistent organizations (**B**, **C**, and **D**). Absent species appear in gray. Inactive reactions and interactions are dashed. Panel **B **represents Organization 10 = {*A*, *I*} where inhibitor *I *represses both reactions from *A *to *P*1 and *P*2. Panels **C **and **D **represent Organization 11 = {*A*, *P*1} and Organization 12 = {*A*, *P*2} where one pathway is active, either over *P1 *or *P2*.

As an example for the presented procedure, we analyze a simple reaction network comprising – apart from inflow and outflow – two reactions forming a switch as depicted in Figure 1(A). The product of one reaction inhibits the other reaction and vice versa. Additionally, an inhibitor *I *can shut down both reactions. Thus, we have a simple *AND *function that requires for both reactions that both *I and P*1, respectively *P*2, are absent. A model without regulation would contain only reactions transforming *A *to *P1 *and *P2*, the influx to *A *and the outflux from the products: R' = {∅ → *A*, *A *→ *P*1, *A *→ *P*2, *P*1 → ∅, *P*2 → ∅}.

The boolean expressions describing the regulation are:

*A *→ *P*1 if ¬*I *⋀ ¬*P*2

*A *→ *P*2 if ¬*I *⋀ ¬*P*1.

Applying the presented procedure, these expressions are transformed into chemical reactions. The resulting reaction network contains the following reactions:

R = {

(1a) ∅ → *A*,

(2a) $$\bar{P2}+\bar{I}+A\to \bar{P2}+\bar{I}+P1,$$

(3a) $$\bar{P1}+\bar{I}+A\to \bar{P1}+\bar{I}+P2,$$

(4) *P*1 → ∅

(5) *P*2 → ∅ }.

The network contains 16 organizations as listed in Table 1.

**Table 1:** A simple switch. All organizations of the regulatory switch network (Figure 18.1). Three organizations are consistent: 10, 11, and 12 (marked bold).

Org.	Species	Real Species
1	*A*	-
2	*A*, *Ī*	-
3	*A*, *I*	-
4	*A*, $\bar{P2}$	-
5	*A*, $\bar{P1}$	-
6	*A*, *I*, *Ī*	-
7	*A*, *I*, $\bar{P1}$	-
8	*A*, *I*, $\bar{P2}$	-
9	*A*, $\bar{P1}$, $\bar{P2}$	-
**10**	*A*, *I*, $\bar{P1}$, $\bar{P2}$	*A*, *I*
**11**	*A*, *Ī*, *P*1, $\bar{P2}$	*A*, *P*1
**12**	*A*, *Ī*, $\bar{P1}$, *P*2	*A*, *P*2
13	*A*, *Ī*, *I*, $\bar{P1}$, $\bar{P2}$	-
14	*A*, *Ī*, *I*, *P*1, $\bar{P2}$	-
15	*A*, *Ī*, $\bar{P1}$, *P*1, $\bar{P2}$, *P*2	-
16	*A*, *Ī*, *I*, $\bar{P1}$, *P*1, $\bar{P2}$, *P*2	-

Three organizations are consistent: *O*_10 _= {*A*, *I*, $\bar{P1}$, $\bar{P2}$}, *O*_11 _= {*A*, *Ī*, *P*1, $\bar{P2}$}, and *O*_12 _= {*A*, *Ī*, $\bar{P1}$, *P*2}. In the remaining organizations it is at least for one species not clearly defined whether it is present or not. Taking Organization 2 for example, the presence of *A *and *I *is determined with *A *present and *I *absent, but there is no information concerning species *P*1 and *P*2. In Organization 6, inhibitor *I *is present and absent at the same time. Figure 1(B,C,D)) depicts the reaction networks belonging to the three consistent organizations. They represent the three states of the switch. In Organization 10, inhibitor *I *is present and shuts down reactions (2) and (3), turning the switch off. In the other two consistent organizations *I *is not present and there is either a flux through reaction (2) (Organization 11) or through reaction (3) (Organization 12). They represent the two other states of the switch.

## Results

We apply the method to a model of the central metabolism of *E. coli *by Covert and Palsson [19]. The authors used the model to study the effects of regulation on flux balance analysis. The regulatory network is defined by a set of boolean functions. There are 73 enzymes, which catalyze 113 reactions. Of these reactions, 43 are regulated by 16 regulatory proteins and therefore controlled by logic statements. The unregulated proteins are assumed to be present in the cell at all times. We add an inflow for all of them in our analysis. To incorporate the regulation into the reaction network, we add the proteins that catalyze reactions explicitly as catalysts in the reactions as described above. Following the introduced protocol, the regulatory logic is incorporated by introducing pseudo species and adapting the reactions accordingly. The activity of several genes is described by boolean statements. Appropriate chemical reactions are added to model this gene regulation. We analyze two variants of the network model by Covert and Palsson [19]: a simplified *core network *to study wildtype growth phenotypes on different carbon sources, and the *complete network *for predicting knockout experiments. Both networks are listed in the Supplement (see Additional file 1) and provided as SBML files (see Additional files 2 and 3).

### The core network model

For studying growth on different carbon sources including diauxic shift, the network is reduced to the set of reactions that lead from external glucose, lactose, and glycerol to pyruvate via glycolysis. Additionally, the pentose-phosphate pathway reactions and the reactions leading from glucose-6-phosphate to this pathway are removed. The resulting network comprises 49 reactions of the original network. The considered part of the network does not contain any ATP production. However, ATP is used up by some reactions, for example in glucose uptake. Therefore, ATP is provided as input. Furthermore, UTP, NAD, NADP, Ubiquinone, and external hydrogen ions are necessary for other uptake and transformation reactions and cannot be provided by this part of the network. These species are added as input as well. To model growth, an outflow is added for every biomass precursor, as in the original network. Since we consider proteins as being active only when they are produced, an outflow for every protein is added, modeling degradation. In order to model different growth media and conditions, self-replicator reactions for external glucose, lactose, glycerol, and oxygen are added of the form *M *→ 2 *M*. These reactions ensure that a constant supply of the respective species is available, whenever it is considered to be present. Using self-replicator reactions, all 2^4 ^= 16 different growth conditions can be modeled in a single network and can be simultaneously considered in one analysis.

The final model comprises 95 species (including 15 pseudo species representing the absence of a species) and 168 reactions. The complete list of network reactions can be found in the Supplement.

### The complete network model

For predicting the lethality of gene knockouts we use the complete network model of the regulated central metabolism of *E. coli *by Covert and Palsson [19]. Depending on the availability of oxygen and the different carbon sources in the growth medium, influxes are added for the respective external species. The currency metabolites HEXT, PI, ADP, ATP, NAD, NADH, Q, QH2, NADP, NADPH, FAD, FADH, UTP, and COA are considered to be unconditionally available in the cell. Input reactions are added for all these species. Without the influxes of external carbon sources and oxygen, the network contains 227 species and 468 reactions.

#### Growth Phenotypes on Different Carbon Sources

The core network model contains 16 consistent organizations. They are listed in Table 2 (see Supplement for a graphical representation). The consistent organizations coincide with the 16 possible growth conditions. The smallest organization, Organization 1, just contains the input metabolites plus the products of the hydrolyzation of ATP, ADP, and phosphate. When analyzing the genes that are active in this organization, we find that the response regulators for glucose, lactose, and glycerol are active, indicating that the respective carbon sources are not present. Due to the absence of oxygen, the aerobic response regulators ArcA and Fnr are also active.

**Table 2:** Growth phenotypes of the core model. Consistent organizations in the core network model of the regulated central metabolism of *E. coli*, ordered by size.

Consistent Organiza.	Species	Growth medium	Uptake
1	Input metabolites, ADP, PI, ArcA, Fnr, GalR, GalS, GlpR, LacI, Mlc, PykF, Ubiquitous proteins	-	-
2	Input metabolites, ADP, O2, O2xt, PI, GalR, GalS, GlpR, LacI, Mlc, PykF, Ubiquitous proteins	O2	-
3	Input metabolites, Glycolysis metabolites, ADP, G1P, GLC, GLCxt, LCTSxt, NADH, PI, PPI, UDPG, ArcA, Crr, FadR, Fnr, Food, GalP, GalR, GalS, GlpR, LacI, Pgk, PtsGHI, PykF, Ubiquitous proteins	GLC	GLC
4	Input metabolites, Glycolysis metabolites, ADP, G1P, GLC, GLCxt, LCTSxt, NADH, O2, O2xt, PI, PPI, UDPG, ArcA, Crr, FadR, Fnr, Food, GalP, GlpR, Pgk, PtsGHI, PykF, Ubiquitous proteins	GLC, LCTS	GLC
5	Input metabolites, Glycolysis metabolites, ADP, G1P, GLC, GLCxt, GLxt, LCTSxt, NADH, PI, PPI, UDPG, ArcA, Crr, FadR, Fnr, Food, GalP, GalR, GalS, LacI, Pgk, PtsGHI, PykF, Ubiquitous proteins	GLC, GL	GLC
6	Input metabolites, Glycolysis metabolites, ADP, G1P, GLC, GLCxt, GLxt, LCTSxt, NADH, O2, O2xt, PI, PPI, UDPG, ArcA, Crr, FadR, Fnr, Food, GalP, Pgk, PtsGHI, PykF, Ubiquitous proteins	GLC, GL, LCTS	GLC
7	Input metabolites, Glycolysis metabolites, ADP, G1P, GLC, GLCxt, NADH, PI, PPI, UDPG, Crr, FadR, Food, GalP, GalR, GalS, GlpR, LacI, Pgk, PtsGHI, PykF, Ubiquitous proteins	GLC, O2	GLC
8	Input metabolites, Glycolysis metabolites, ADP, G1P, GLC, GLCxt, NADH, O2, O2xt, PI, PPI, UDPG, Crr, FadR, Food, GalP, GlpR, Pgk, PtsGHI, PykF, Ubiquitous proteins	GLC, LCTS, O2	GLC
9	Input metabolites, Glycolysis metabolites, ADP, G1P, GLC, GLCxt, GLxt, NADH, PI, PPI, UDPG, Crr, FadR, Food, GalP, GalR, GalS, LacI, Pgk, PtsGHI, PykF, Ubiquitous proteins	GLC, GL, O2	GLC
10	Input metabolites, Glycolysis metabolites, ADP, G1P, GLC, GLCxt, GLxt, NADH, O2, O2xt, PI, PPI, UDPG, Crr, FadR, Food, GalP, Pgk, PtsGHI, PykF, Ubiquitous proteins	GLC, GL, LCTS, O2	GLC
11	Input metabolites, Glycolysis metabolites, ADP, G1P, GL, GL3P, GLxt, NADH, NADPH, O2, O2xt, PI, PPI, QH2, UDPG, ArcA, Crr, Fnr, Food, GalP, GalR, GalS, GlpABC, GlpF, GlpK, LacI, Mlc, Pgk, PtsGHI, PykF, Ubiquitous proteins	GL	GL
12	Input metabolites, Glycolysis metabolites, ADP, G1P, GL, GL3P, GLxt, NADH, NADPH, PI, PPI, QH2, UDPG, Crr, Food, GalP, GalR, GalS, GlpD, GlpF, GlpK, LacI, Mlc, Pgk, PtsGHI, PykF, Ubiquitous proteins	GL, O2	GL
13	Input metabolites, Glycolysis metabolites, Lactose derivatives, ADP, G1P, GLC, LCTS, LCTSxt, NADH, PI, PPI, UDPG, ArcA, Crr, Fnr, Food, GalE, GalK, GalM, GalP, GalT, GlpR, LacY, LacZ, Mlc, Pgk, PtsGHI, PykF, Ubiquitous Proteins	LCTS	LCTS
14	Input metabolites, Glycolysis metabolites, Lactose derivatives, ADP, G1P, GLC, LCTS, LCTSxt, NADH, O2, O2xt, PI, PPI, UDPG, ArcA, Crr, Fnr, Food, GalE, GalK, GalM, GalP, GalT, LacY, LacZ, Mlc, Pgk, PtsGHI, PykF, Ubiquitous Proteins	GL, LCTS	LCTS
15	Input metabolites, Glycolysis metabolites, Lactose derivatives, ADP, G1P, GLC, GLxt, LCTS, LCTSxt, NADH, PI, PPI, UDPG, Crr, Food, GalE, GalK, GalM, GalP, GalT, GlpR, LacY, LacZ, Mlc, Pgk, PtsGHI, PykF, Ubiquitous proteins	LCTS, O2	LCTS
16	Input metabolites, Glycolysis metabolites, Lactose derivatives, ADP, G1P, GLC, GLxt, LCTS, LCTSxt, NADH, O2, O2xt, PI, PPI, UDPG, Crr, Food, GalE, GalK, GalM, GalP, GalT, LacY, LacZ, Mlc, Pgk, PtsGHI, PykF, Ubiquitous Proteins	GL, LCTS, O2	LCTS

For brevity, pseudo species indicating the absence of a species are not listed. A list of abbrevations can be found in the Supplement. A species followed by 'xt' denotes its extra-cellular form. "Ubiquitous proteins" include the proteins that are considered ubiquitously present in the cell and therefore are not listed separately. They are: Eno, Fba, Fbp, GalU, GapA, Glk, GpmA, GpmB, GpsA, PfkA, PfkB, Pgi, Pgm, PykA, and TpiA. "Input metabolites" denotes the metabolites provided as input to the system: HEXT (external hydrogen), Q (Ubiquinone), ATP, UTP, NAD, and NADP. "Glycolysis metabolites" denotes the metabolites of the glycolysis: G6P, F6P, FDP, T3P2, T3P1, 13PDG, 3PG, 2PG, PEP, and PYR. "Lactose derivatives" denotes the derivatives of lactose in the central metabolism: GAL1P, GLAC, UDPGAL, bDGLAC, bDGLC.

In Organization 2, external oxygen is available. Consequently, the aerobic response regulators ArcA and Fnr are absent here. This is the only difference between Organizations 1 and 2.

#### Glucose uptake

The first organization that utilizes an external carbon source is Organization 3, which contains the reactions for glucose uptake. Consequently, the metabolites of the central metabolism are present in this organization. When examining the proteins of the organization, we find that the glucose response regulator Mlc is absent. The organization next in size is Organization 4. Here, lactose is additionally in the medium. Although the repressor of the *lac *gene, LacI, is absent in the organization, no uptake reactions for external lactose are contained in the organization. The lactose permease LacY, a product of the *lac *genes, is missing. As glucose is available in the medium, the lactose uptake system is not induced by the presence of external lactose. This effect is known as inducer exclusion. The metabolite required for upregulation of the *lac *genes is not taken up by the cell. Organization 5 represents a similar case in which glycerol is available in the growth medium but not taken up. All external carbon sources and oxygen are available in Organization 10, but the cell is still exclusively utilizing glucose. Organizations 6 to 9 represent further input combinations defining growth conditions with external glucose available. The availability of oxygen does not change the reactions in the part of the central metabolism that is considered in the core model.

#### Lactose uptake

In Organization 13, lactose is the exclusive external carbon source. Consequently, LacI is absent as it is bound by allolactose, a derivative of lactose. Hence, it cannot repress the genes necessary for lactose uptake and utilization. We find the corresponding gene products present in this organization, namely LacZ and LacY. Additionally, derivatives of lactose, for example galactose, are contained in this organization. These metabolites are created in the pathway leading from lactose to the central metabolism. Another diauxic shift effect can be observed in Organization 14. Here, external lactose and glycerol are present as carbon sources, but as in the case with glucose and lactose, only lactose is taken up. Organizations 15 and 16 represent further growth conditions in which lactose is taken up. Once again, the availability of oxygen does not change the reactions in the modeled part of the central metabolism.

#### Glycerol uptake

Glycerol is the exclusive external carbon source in Organization 11. As all proteins necessary for glycerol uptake are present, glycerol is taken up. For glycerol uptake, three different enzymes catalyze the reaction from glycerol-3-phosphate to dihydroxyacetone-phospate, a metabolite of glycolysis. One of these enzymes, glycerol-3-phosphate-dehydrogenase, is constitutively expressed in the model. The other two proteins, glycerol-3-phosphate kinases GlpABC and GlpD are specific for anaerobic, respectively aerobic growth conditions. Therefore, GlpABC is present and GlpD absent in Organization 11, where no oxygen is available. When oxygen is available as in Organization 12, GlpD is present and GlpABC absent.

#### Predicting Gene Knockout Experiments

Knockout experiments are performed using the complete network model. Gene knockouts are modeled by deleting all reactions in which the corresponding protein takes part as educt or product. The set of consistent organizations is determined for each knockout experiment using a heuristic approach detailed in the Supplement. The lethality of a knockout can be predicted by the existence of organizations containing all biomass precursor metabolites. If such an organizations is not found, the knockout is predicted to be lethal. We use organization theory (OT) and an adapted version of organization theory (aOT, see below) to predict the same gene knockouts as Covert and Palsson [19], who used regulatory flux balance analysis (rFBA) for gene knockout predictions. Reference [19] is also our source for *in vivo *data and predictions by flux balance analysis (FBA) and rFBA. The results are summarized in Table 3. Out of 116 experiments, the predictions by FBA are correct in 97 cases (83,6%). The predictions by rFBA are correct in 106 cases (91,4%) and improve the results of FBA in nine cases. Unmodified OT predicts the lethality of knockouts correctly in 101 cases (87,1%), while aOT predicts 106 cases (91,4%) correctly as rFBA. The additional model-specific assumptions made by aOT are taken from Covert and Palsson [19] and will be described in detail now.

**Table 3:** Comparison of knockout predictions.

	**glc**	**gl**	**suc**	**ac**	**rib**	**glc (-O_2_)**	**Dual Substr**.	**Reference**
*aceA*	+/+/+/+		+/+/+/+	-/-/-/-		+/+/+/+		[30]
*aceB*				-/-/-/-				[31]
*aceEF*	-/+/-/-		-/+/-/-	+/+/+/+		+/+/+/+	+/+/+/+ (glc-ac)	[32]
*ackA*				**+/+/+/-**				[33]
*ackA *+ *pta *+ *acs*				-/-/-/-				[33]
*acnA*	+/+/+/+	+/+/+/+	+/+/+/+	+/+/+/+		+/+/+/+		[31,34]
*acnB*	+/+/+/+	+/+/+/+	+/+/+/+	-/+/+/+		+/+/+/+		[34]
*acnA *+ *acnB*	-/-/-/-	-/-/-/-	-/-/-/-	-/-/-/-		-/-/-/-		[34]
*acs*				+/+/+/+				[33]
*adh*	+/+/+/+					-/+/+/+		[35]
*cyd*	+/+/+/+							[36]
*cyo*	+/+/+/+							[36]
*eno*	-/-/-/-	-/-/-/-	-/-/-/-				+/+/+/+ (gl-suc)	[37]
*fbaA*	-/+/+/+							[38]
*fbp*	+/+/+/+	-/-/-/-	-/-/-/-	-/-/-/-				[39]
*frdA*	+/+/+/+		+/+/+/+	+/+/+/+		+/+/+/+		[30]
*fumA*				-/+/-/-		+/+/+/+		[31]
*gap*	-/-/-/-	-/-/-/-	-/-/-/-				+/+/+/+ (gl-suc)	[37]
*glk*	+/+/+/+							[38]
*glk *+ *pfkA*	+/+/+/+							[38]
*glk *+ *pts*	-/-/-/-							[38]
*gltA*	-/-/-/-			-/-/-/-				[34]
*gnd*	+/+/+/+							[38]
*icd*	-/-/-/-			-/-/-/-				[34]
*mdh*	+/+/+/+	+/+/+/+	+/+/+/+			+/+/+/+		[40]
*ndh*	+/+/+/+	+/+/+/+						[41]
*nuo*	+/+/+/+	+/+/+/+						[41]
*pfl*						+/+/+/+		[42]
*pgi*	+/+/+/+	+/-/-/-	+/-/-/-					[38]
*pgi *+ *gnd*	-/-/-/-							[38]
*pgi *+ *zwf*	-/-/-/-							[38]
*pgk*	-/-/-/-	-/-/-/-	-/-/-/-				+/+/+/+ (gl-suc)	[37]
*pgl*	+/+/+/+							[38]
*ppc*	-/+/-/-	**-/+/-/+**	+/+/+/+				+/+/+/+ (gl-suc) +/+/+/+ (glc-suc)	[38,40]
*pta*				**+/+/+/-**				[33]
*pts*	+/+/+/+							[38]
*pykA*	+/+/+/+							[38]
*pykA *+ *pykF*	+/+/+/+							[38]
*pykF*	+/+/+/+							[38]
*rpiA*	**-/+/-/+**				+/+/+/+		+/+/+/+ (glc-rib)	[43]
*rpiA *+ *rpiB*	**-/-/-/+**				-/+/+/+		+/+/+/+ (glc-rib)	[43]
*rpiB*	+/+/+/+				+/+/+/+		+/+/+/+ (glc-rib)	[43]
*rpiR *+ *rpiA*	+/N/+/+				+/N/+/+		+/N/+/+ (glc-rib)	[43]
*sdhABCD*	+/+/+/+		-/-/-/-	-/-/-/-		+/+/+/+		[30]
*sucAB- lpd*	-/+/+/+		-/+/+/+	-/+/+/+		+/+/+/+	+/+/+/+ (glc-suc)	[30,32]
*tpi*	-/+/+/+	-/-/-/-	-/-/-/-	-/-/-/-			+/+/+/+ (glc-suc) +/+/+/+ (glc-gl)	[37,44]
*zwf*	+/+/+/+							[38]

Comparing *in vivo *knockout experiment results with predictions made by FBA, rFBA, OT and by aOT. A '+' indicates growth, a '-' no growth of the mutants on the indicated substrate(s). For cases denoted as 'N', data was not available. Results and predictions are derived from *in vivo*/FBA/rFBA/(a)OT. *In vivo *data and references, FBA and rFBA predictions are taken from Ref. [19]. In five instances, predictions made by OT deviate from rFBA predictions (bold cases). See text for discussion. The growth medium contained glucose (glc), glycerol (gl), succinate (suc), acetate (ac), or ribose (rib). Anaerobic condition is denoted by '-O_2_'.

#### Assumption that accumulation of mass is lethal

In two cases, OT predicts a lethal knockout to be nonlethal (*rpiA*, and *rpiA *+ *rpiB *on glucose). The self-maintenance property allows for the accumulation of internal metabolites, while in rFBA, only steady states are considered, and any accumulation of metabolites is regarded as lethal. In these two cases, the organizations containing all biomass precursors contain metabolites with positive productions. (Note, that all species except the pseudo species indicating the absence of species decay in the network model. Hence, all organizations are indeed balanced organizations. However, accumulation of metabolites occur, if the decay reactions, which are not present in the original network, are removed.) Hence, OT predicts the knockout to be nonlethal while rFBA predicts it to be lethal as no steady state exists. In aOT we restrict our analysis to organizations that are balanced (*i.e*., internal metabolites are not allowed to accumulate). With this model specific assumption aOT classifies the two knockout experiments correctly.

#### Assumption that secreted molecules have no effect

Further three incorrect predictions by OT (*ackA *and *pta *on acetate, and *ppc *on glycerol) yield deeper insights into the differences between chemical organization theory and regulatory flux balance analysis, namely in the treatment of by the cell secreted molecules.

**Figure 2:**
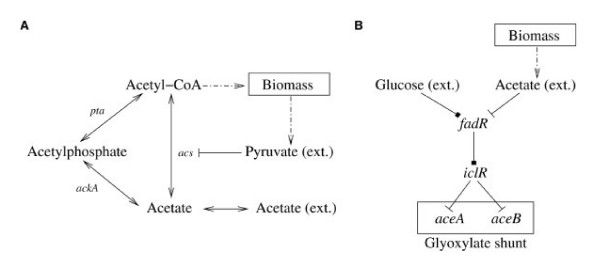
**Illustration of knockout experiments**. Panel **A**: Illustration of the sub-network that explains why deletion of *pta *or *ackA *is wrongly predicted as lethal by OT. According to the model, the alternative route via *acs *is inhibited by the presence of external pyruvate, which is always excreted when biomass is produced. Thus, there cannot be an uptake of external acetate. *In vivo*, however, the excreted pyruvate is negligible. Panel **B**: Illustration of the sub-network that explains why deletion of *ppc *is wrongly predicted as viable by OT. The glyoxylate shunt is activated if no external glucose but external acetate is present. According to the model, acetate is always excreted when biomass is produced. Therefore, the glyoxylate shunt is always upregulated, if glucose is not contained in the growth medium. Arrows indicate metabolic reactions, squares indicate activation and T-shaped lines inhibition. Dotted arrows indicate schematic reactions, which abstract a set of metabolic reactions.

In the case of acetate uptake, there are two pathways that enable the utilization of this carbon source as depicted in Figure 2(A). One pathway leads directly from acetate to acetyl-CoA, and the other takes the route via acetyl phosphate. The first pathway is catalyzed by the acetyl-CoA synthethase (gene *acs*). According to the model, the corresponding gene is only transcribed if no carbon source is available or at most acetate or formate, or both. The second pathway is catalyzed by acetate kinase A (gene *ackA*) and phosphotransacetylase (gene *pta*). If one of these genes is knocked out, the first pathway can still support the central metabolism, given that acetate is the exclusive external carbon source. In this case, chemical organization theory predicts both knockouts as lethal, which is not the case *in vivo *and correctly predicted by rFBA. The reason for this discrepancy is that in any network containing the biomass precursor metabolite pyruvate, this metabolite will be secreted by the cell. Therefore, such a network also comprises the external form of pyruvate which is an inhibitor for the only remaining uptake reaction for acetate. Consequently, there exists no organization containing all biomass precursor metabolites when acetate is the exclusive carbon source in the growth medium and the second pathway is knocked out. Because the presence of metabolites is not explicitly considered in rFBA, this inhibition is not detected by rFBA. However, because the knockout is nonlethal in *in vivo *experiments, the levels of secreted pyruvate might not be suffcient to have an effect on the expression of *acs*. Or, the cell switches its uptake from acetate to pyruvate until it is depleted and then switches back to acetate again.

The incorrect prediction of the knockout of *ppc *on glycerol as nonlethal can be explained by the same argument. Gene *ppc *codes for the phosphoenolpyruvate carboxylase, which supplies the citric acid cycle with oxaloacetate (OA). When *ppc *is knocked out, the only alternative for OA production is the glyoxylate shunt, consisting of the isocitrate lyase (gene *aceA*) and the malate synthase A (gene *aceB*). However, the glyoxylate shunt is only active if *E. coli *grows on acetate or fatty acids as the sole carbon source [21]. Hence, the knockout of *ppc *on a glycerol growth medium is lethal *in vivo*, as the glyoxylate shunt is not activated. In the model, the glyoxylate shunt is regulated by the fatty acid and acetate response regulators IclR and FadR as depicted in Figure 2(B). Gene *fadR *is only expressed if external glucose, or no acetate is available in the growth media. If activated, FadR leads to an upregulation of *iclR*, which then leads to a downregulation of *aceA *and *aceB*, inactivating the glyoxylate shunt. If acetate is available in the growth medium, *fadR *is not expressed, and thus *aceA *and *aceB *are expressed at high levels, activating the glyoxylate shunt. Any organization containing the biomass precursor metabolite acetyl-CoA also contains acetate, which is secreted. Hence, any organization containing the biomass precursors also contains the external form of acetate, which activates the glyoxylate shunt.

Even though considerable amounts of acetate secretion on glycerol media has only been reported in high density cultures [22], OT indicates the possibility for *E. coli *to grow on glycerol if *ppc *is knocked out. Our result suggests that the activation of the glyoxylate shunt would be enough. There have been reports of *E. coli *strains growing on glucose media that were also able to grow with *ppc *knocked out, which is usually lethal. This was facilitated by a mutation that lead to an upregulation of the glyoxylate shunt [23]. Thus, the results of OT might be biological feasible in this case.

The problems encountered in the regulation by secreted species can be resolved by introducing a pseudo species representing the secreted version of a species. Therefore, in aOT, a metabolite that can be secreted is represented by two species: one represents the metabolite at high concentration (*e.g*., when externally supplied), the other species represents the metabolite at low concentration (*e.g*., when secreted). These modifications allow aOT to correctly predict the three cases discussed above. Note that the underlying assumption of this modification is also made by Covert and Palsson [19].

## Discussion

By transforming the boolean formalism that represents the regulation of a metabolic network into reaction rules, we were able to demonstrate how chemical organization theory can be applied to regulated metabolic networks. Using a model of the central metabolism of *E. coli *[19], each of the 16 wildtype growth scenarios were correctly predicted down to the presence of each protein. Each external condition could be directly mapped to a single organization implying a distinct qualitative state of the network (*i.e*., a set of molecular species present).

### Knockout experiments

Without specific assumptions, organization theory (OT) was able to predict the lethality of knockout experiments correctly in 101 out of 116 cases (87.1%). With model specific assumptions, "adapted" organization theory (aOT) was able to predict the lethality of knockout experiments correctly in 106 cases (91.4%), achieving the same performance as rFBA [19]. When comparing the performance of OT and rFBA with respect to knockout predictions, it must be noted that the model used for the comparison was co-developed with rFBA. Specific assumptions were made that were deliberatly not made in the pure organization analysis as OT shall provide an universal analysis technique applicable to general biological network models (*e.g*., [24]). In this light, we consider the performance of organization theory as competitive.

Moreover, there are cases in other models in which predictions by OT are more accurate than those by FBA. This occurs, for example, due to the fact that FBA only incompletely takes co-factors into account. These co-factors are molecules that are necessary for some reactions to proceed. They can interact through various means with the substrates and products of a reaction. In the (unregulated) metabolic model by Reed et al. [25], we identified 10 cases in which OT correctly classifies a knockout as lethal while (r)FBA does not. The reason for this difference is that the identified co-factors are necessary for producing biomass metabolites (i.e., metabolites that appear on the left hand side of the biomass producing reaction), but they are not considered as biomass metabolites (see Supplement for details).

### Influence of model specific assumptions

In our analysis of the model by Covert and Palsson [19], we found five cases in which OT predictions differ from rFBA predictions. All of these cases can be resolved by aOT in a straightforward way by taking assumptions into account also made by rFBA in [19]. In particular, the deviation between OT and (r)FBA has uncovered two critical aspects:

First, (r)FBA only considers steady states. A system state with accumulating metabolites is regarded as lethal. In OT, accumulating metabolites are explicitly allowed to also cover system states related to growth. To adopt the steady state assumption in OT however, one simply can restrict the analysis to balanced organizations in aOT. However, only considering steady states is not necessarily the best "natural" choice. As models usually are not complete, the biological system might contain pathways that are not modeled but can take care of overproduced metabolites. Also, certain molecular species accumulate in the cell at certain time points, for example in different phases of the cell cycle. Hence, states with positive productions of certain species are not necessarily lethal.

Second, in OT only the presence or absence of metabolites is considered. Hence, even smallest concentrations of species will potentially trigger further responses. When secreted metabolites have much lower concentrations than external metabolites supplied by the growth medium, this can lead to wrong predictions as secreted metabolites shall not trigger further regulation (cf. Figure 2). However, the problem can easily be resolved by introducing two species representing the metabolite at a high and at a low concentration, respectively. Alternatively, we could use the positive flow from the carbon source as a signal for the high concentration of the external metabolite (as it has been done by Covert and Palsson [19]). Note however, that with the unmodified OT we found a phenotype that indicates how to bypass the *in vivo *lethality of a knockout in one case.

### Identification of all potential qualitative phenotypes

Organization theory provides a rigorous link between an organization and the potential dynamics of the reaction system (cf. Theorem 1 in Ref. [5]): if there is a steady state, then the species with positive concentrations must be an organization. Thus, we can guarantee that there is no other species combination that can give rise to a stationary state. Note that the species sets our method identifies contain also proteins, so that the organizations we obtain describe not only which metabolites are present but also which proteins are active in any possible steady state.

A related issue has been raised by Shlomi et al. [26]: The choice of a flux vector producing biomass in the FBA phase of rFBA leaves open a whole range of possible flux vectors in the space of possible solutions of rFBA. Thus, also the outcome of the experiments might depend on this choice. In contrast, OT takes all possible fluxes into account. If there are several qualitative phenotypes (i.e. sets of species with positive concentrations) consistent with the regulatory rules, several organizations will be found (see Methods).

### Application to large-scale models

The largest organization does not necessarily encompass the whole system. Thus when analyzing larger models, OT allows the exact prediction of those parts of a model that can give rise to a steady state. Parts missing in such a state can then be refined. For example in the case of knockout experiments, we can determine which part of a network is still available. Such knowledge is important when trying to reduce a metabolic model for a specific purpose, for example to increase the production of a certain metabolite. Even though the presented method can also be used for genome scale networks, it remains to be seen if all of the regulated organization in such a system could still be found. The bottleneck in the computation is the determination of all organizations of a regulated network before checking if they are consistent. Their number can grow exponentially with network size, in which case computation is primarily constrained by the available memory. This problem might be circumvented by an approach that partitions the solution space of the self-maintainance condition and searches those partitions in parallel for organizations (see Supplement for algorithmic details). However, such an approach has not yet been implemented. Currently, the analysis of a pure (non-regulated) metabolic network of genome scale by [25] is possible with the heuristic approach (see Supplement). If the number of organizations in such a network is small, all of them can be found with the heuristic approach [27]. Whether and how this scales to regulated genome-scale models is an open issue.

### Other Approaches for Integrated Network Analysis

In recent years other approaches for the integration of regulatory networks into stoichiometric analysis have been proposed: Steady-state regulatory flux balance analysis (SR-FBA) [26] builds on rFBA and allows the flux analysis within one step. Boolean regulatory rules are integrated into the FBA approach by using mixed-integer linear programming. Thus, fluxes that obey the regulatory constraints can be computed directly without prior determination of the state of the regulatory network as in rFBA. In contrast to our approach SR-FBA focuses, like FBA, on specific fluxes through the network. Thus a possible flux for each possible reaction among the molecules like in organization theory (by the definition of closure), is not required for a feasible flux vector in SR-FBA. Likewise problems with co-factors like in FBA occur (see Discussion and Supplement).

Another approach, a matrix formalism to analyze regulatory systems, has been proposed by Gianchandani et al. [28]. Similar to our approach, they formulate the regulatory network by representing it as reactions in the stoichiometric matrix. Then they analyze the integrated network by using extreme pathway analysis [29]. The main difference to our work is that modelling based on the stoichiometric matrix requires a flux through the regulatory network. Thus inhibitors and activators are consumed upon interaction and are not modelled as catalysts. Even though the authors only analyzed a pure regulatory network, an integration into a metabolic network seems to be possible. Interestingly, the concept of a functional state of the resulting network, *i.e*., the state when all external inputs are defined, resembles the organizations we found. However, those states are restricted to the regulatory part of the network, since the closure of the participating molecules is not taken into account. Another aspect of this concept that has not yet been analyzed is to which extent this method can also be applied to larger networks, for example those we analyzed in this work. The integration of the regulatory network into a metabolic network using a flux through the network might further increase the combinatorial explosion of the number of extreme pathways. Nonetheless, this approach is valuable for identifying underlying pathways in a regulatory network, a prospect which has not yet been analyzed in connection with OT.

## Conclusion

Because OT does not rely on kinetics, it can serve as a first step to analyze the potential behavior of regulated systems. The analysis delivers all potential network phenotypes described by the sets of molecular species that can coexist over a long time (cf. Theorem 1 in Ref. [5]). The further analysis of the network can then focus on interesting phenotypes. Taking the other direction, it is possible to validate *in silico *network models. All phenotypes of interest observed *in vivo *should have corresponding organizations in the network model.

When regulation is considered in metabolic networks, the presented approach offers the advantage that both the metabolism and its regulation are modeled within one single framework: chemical reaction rules forming a network. The unification comes at the expense of introducing a set of pseudo species to represent the absence of species. This allows one to model and consider inhibitory interactions within the framework of organization theory. Using an appropriate user interface, the pseudo species can be easily hidden.

## Authors' contributions

CK and PD introduced the idea of pseudo species indicating the absence of metabolites. All authors contributed to the presented new concepts and procedures building on the framework of organization theory. Software implementation, data preparation, and analysis was done by CK and FC. All authors read and approved the final manuscript.

## Supplementary Material

Additional file 1 Supplement including the analyzed network models and a descritption of the employed algorithms to compute organizations. Click here for file

Additional file 2 The analyzed core model in SBML format. Click here for file

Additional file 3 The analyzed complete model in SBML format. Click here for file
