# Supplementary material for: Phenotype prediction in regulated metabolic networks
Source: BMC Syst Biol. 2008 Apr 25;2:37. doi: 10.1186/1752-0509-2-37 (PMC2443871; doi:10.1186/1752-0509-2-37)
Supplement: Additional file 1 — Supplement including the analyzed network models and a descritption of the employed algorithms to compute organizations. [file 1752-0509-2-37-S1.pdf]

Phenotype Prediction in Regulated Metabolic Networks
-Supplementary Material-

Christoph Kaleta, Florian Centler, Pietro Speroni di Fenizio, Peter Dittrich

April 3, 2008

Bio Systems Analysis Group
Department of Mathematics and Computer Science
Friedrich-Schiller-University Jena
D-07737 Jena, Germany
Jena Centre for Bioinformatics (JCB), Jena, Germany

Contents

1 Organizations in the Core Network Model of *E. coli* **2**

2 The Regulated *E. coli* Network **4**
2.1 Metabolites 4
2.2 Genes and Proteins 5
2.3 Pseudo Species 6
2.4 Spontaneously Created Species 6
2.5 Spontaneously Decaying Species 6
2.6 Transport Reactions 6
2.7 Metabolic reactions 7
2.8 Regulatory Reactions 9

3 Analysis of a Genome-Scale Metabolic Model **11**
3.1 Role of Co-factors 11
3.1.1 Problem of Non-Metabolic Species 11
3.1.2 Problem of Redundant Co-factors 11

4 A Flux Based Algorithm for Computing Organizations **12**
4.1 Using the self-maintenance cone in flux-space 12
4.2 The extreme ray algorithm 12
4.2.1 Step 1: computing elementary organizations 13
4.2.2 Step 2 & 3: computing all organizations 13
4.3 An heuristic approach to organization computation 14

1 Organizations in the Core Network Model of *E. coli*

Cons. Org.	1	2	3	4	5	6	7	8	9	10	11	12	13	14	15	16
Uptake			GLC								GL		LCTS			
GLCxt			■	■	■	■	■	■	■	■						
LCTSxt				■		■		■		■			■	■	■	■
GLxt					■	■			■	■	■	■		■		■
O2xt		■					■	■	■	■		■			■	■
Input metabolites	■	■	■	■	■	■	■	■	■	■	■	■	■	■	■	■
Ubiquitous proteins	■	■	■	■	■	■	■	■	■	■	■	■	■	■	■	■
Glycolysis metabolites			■	■	■	■	■	■	■	■	■	■	■	■	■	■
LCTS derivatives													■	■	■	■
GalEKMT													■	■	■	■
pykF	■	■	■	■	■	■	■	■	■	■	■	■	■	■	■	■
ptsGHI			■	■	■	■	■	■	■	■	■	■	■	■	■	■
ppsA																
pgk			■	■	■	■	■	■	■	■	■	■	■	■	■	■
mlc	■	■									■	■	■	■	■	■
lacZ													■	■	■	■
lacY													■	■	■	■
lacI	■	■	■		■		■		■		■	■				
glpR	■	■	■	■			■	■					■		■	
glpK											■	■				
glpF											■	■				
glpD												■				
glpABC											■					
galS	■	■	■		■		■		■		■	■				
galR	■	■	■		■		■		■		■	■				
galP			■	■	■	■	■	■	■	■	■	■	■	■	■	■
food			■	■	■	■	■	■	■	■	■	■	■	■	■	■
fnr	■		■	■	■	■					■		■	■		
fadR			■	■	■	■	■	■	■	■						
crr			■	■	■	■	■	■	■	■	■	■	■	■	■	■
cra																
arcA	■		■	■	■	■					■		■	■		
UDPG			■	■	■	■	■	■	■	■	■	■	■	■	■	■
QH2											■	■				
PPI			■	■	■	■	■	■	■	■	■	■	■	■	■	■
PI	■	■	■	■	■	■	■	■	■	■	■	■	■	■	■	■
O2		■					■	■	■	■		■			■	■
NADPH											■	■				
NADH			■	■	■	■	■	■	■	■	■	■	■	■	■	■
LCTS													■	■	■	■
GLC			■	■	■	■	■	■	■	■			■	■	■	■
GL3P											■	■				
GL											■	■				
G1P			■	■	■	■	■	■	■	■	■	■	■	■	■	■
ADP	■	■	■	■	■	■	■	■	■	■	■	■	■	■	■	■
AMP																

Table 1: Consistent organizations in the core network model of the regulated central metabolism of *E. coli*, ordered by size. The presence of a metabolite in an organization is indicated by a black box. A list of abbrevations can be found in this Supplement. A species followed by 'xt' denotes its extra-cellular form. "Ubiquitous proteins" include the proteins that are considered ubiquitously present in the cell and therefore are not listed separately. They are: Eno, Fba, Fbp, GalU, GapA, Glk, GpmA, GpmB, GpsA, PfkA, PfkB, Pgi, Pgm, PykA, and TpiA. "Input metabolites" denotes the metabolites provided as input to the system: HEXT (external hydrogen), Q (Ubiquinone), ATP, UTP, NAD, and NADP. "Glycolysis metabolites" denotes the metabolites of the glycolysis: G6P, F6P, FDP, T3P2, T3P1, 13PDG, 3PG, 2PG, PEP, and PYR. "Lactose derivatives" denotes the derivatives of lactose in the central metabolism: GAL1P, GLAC, UDPGAL, bDGLAC, bDGLC.

2 The Regulated *E. coli* Network

Species and reactions marked with '*' make up the core network model. The original network can be found in Covert and Palsson [1]. Due to the incomplete set of reactions, the species responsible for the regulation of *cra* and *pdhR* are not contained in the core network model. They are marked with '†'.

2.1 Metabolites

**Abbr.**	**Metabolite**	**Abbr.**	**Metabolite**
$13PDG^*$	1,3-bis-Phosphoglycerate	$LACxt$	External lactate
$2PG^*$	2-Phosphoglycerate	$LCTS^*$	Lactose
$3PG^*$	3-Phosphoglycerate	$LCTSxt^*$	External Lactose
AC	Acetate	MAL	Malate
$ACCOA$	Acetyl-CoA	NAD^*	Nicotinamide adenine dinucleotide
$ACTP$	Acetyl-phosphate	$NADH^*$	Nicotinamide adenine dinucleotide red.
$ACxt$	External acetate	$NADP^*$	Nicotinamide adenine dinucleotide phosphate
ADP^*	Adenosine diphosphate	$NADPH^*$	Dihydronicotinamide adenine dinucleotide phosphate reduced
AKG	a-Ketoglutarate	$O2^*$	Oxygen
AMP^*	Adenosine monophosphate	$O2xt^*$	External Oxygen
ATP^*	Adenosine triphosphate	OA	Oxaloacetate
$bDGLAC^*$	b-D-Galactose	PEP^*	Phosphoenolpyruvate
$bDGLC^*$	b-D-Glucose	PI^*	Phosphate (inorganic)
$Biomass$	Cell biomass	$PIxt$	External phosphate
CIT	Citrate	PPI^*	Pyrophosphate
$CO2$	Carbon dioxide	PYR^*	Pyruvate
$CO2xt$	External carbon dioxide	$PYRxt$	External pyruvate
COA	Coenzyme A	Q^*	Ubiquinone
$D6PGC$	D-6-Phosphate-gluconate	$QH2^*$	Ubiquinol
$D6PGL$	D-6-Phosphate-glucono-delta-lactone	$R5P$	Ribose 5-phosphate
$E4P$	Erythrose 4-phosphate	RIB	Ribose
ETH	Ethanol	$RIBxt$	External ribose
$ETHxt$	External ethanol	$RL5P$	Ribulose 5-phosphate
$F6P^*$	Fructose 6-phosphate	$S7P$	sedo-Heptulose
FAD	Flavin adenine dinucleotide	$SUCC$	Succinate
$FADH$	Flavin adenine dinucleotide reduced	$SUCCOA$	Succinate CoA
FDP^*	Fructose 1,6-diphosphate	$SUCCxt$	External succinate
FOR	Formate	$T3P1^*$	Glyceraldehyde 3-phosphate
$FORxt$	External Formate	$T3P2^*$	Dihydroxyacetone phosphate
FUM	Fumarate	$UDPG^*$	UDP Glucose
$G1P^*$	Glucose 1-phosphate	$UDPGAL^*$	UDP Galactose
$G6P^*$	Glucose 6-phosphate	UTP^*	Uridine triphosphate
$GAL1P^*$	Galactose 1-Phosphate	$X5P$	Xylulose-5-phosphate
GL^*	Glycerol	$food^*$	carbon source present in medium
$GL3P^*$	Glycerol 3-phosphate	$LactateUP$	lactate uptake activated
$GLAC^*$	Galactose	$CraCondNeg^\dagger$	*cra* regulation
w GLC^*	a-D-Glucose	$ftktA^\dagger$	*cra* regulation
$GLCxt^*$	External glucose	$ftktB^\dagger$	*cra* regulation
GLX	Glyoxylate	$ftalA^\dagger$	*cra* regulation
$Oxid-$	superoxid radicals	$ftalB^\dagger$	*cra* regulation
$Radicals$		$fpgi^\dagger$	*cra* regulation
$GLxt^*$	External glycerol	$PdhRCondNeg^\dagger$	*pdhR* regulation
$HEXT^*$	External H+	$fdctA^\dagger$	*pdhR* regulation
$ICIT$	Isocitrate	$fdcuA^\dagger$	*pdhR* regulation
LAC	D-Lactate	$fdld^\dagger$	*pdhR* regulation

2.2 Genes and Proteins

Gene	Protein	Gene	Protein
aceA	Isocitrate lyase	*pgm**	Phosphoglucomutase
aceB	Malate synthase A	*pntAB*	Pyridine nucleotide transhydrogenase
aceEF, *lpdA*	Pyruvate dehydrogenase	*ppa*	Inorganic pyrophosphatase
ackA	Acetate kinase A	*ppc*	Phosphoenolpyruvate carboxylase
acnA	Aconitase A	*ppsA**	Phosphoenolpyruvate synthase
acnB	Aconitase B	*pta*	Phosphotransacetylase
acs	Acetyl-CoA synthetase	*pykA**	Pyruvate Kinase II
adhE	Acetaldehyde dehydrogenase	*pykF**	Pyruvate Kinase I
adk	Adenylate kinase	*rbsK*	Ribokinase
atpABC− DEFGHI	F0F1-ATPase	*rpe*	Ribulose phosphate 3-epimerase
cydAB	Cytochrome oxidase bd	*rpiA*	Ribose-5-phosphate isomerase A
cyoABCD	Cytochrome oxidase bo3	*rpiB*	Ribose-5-phosphate isomerase B
dld	D-Lactate dehydrogenase 1	*sdhABCD*	Succinate dehydrogenase complex
*eno**	Enolase	*sfcA*	Malic enzyme (NAD)
*fba**	Fructose-1,6-bisphosphatate aldolase	*sucAB*, *lpdA*	2-Ketoglutarate dehyrogenase
*fbp**	Fructose-1,6-bisphosphatase	*sucCD*	Succinyl-CoA synthetase
fdnGHI	Formate dehydrogenase-N	*talA*	Transaldolase A
fdoIHG	Formate dehydrogenase-O	*talB*	Transaldolase B
frdABCD	Fumarate reductase	*tktA*	Transketolase I
fumA	Fumarase A		
fumB	Fumarase B	*tktB*	Transketolase II
fumC	Fumarase C		
*galE**	UDP-glucose 4-epimerase	*tpiA**	Triosphosphate Isomerase
*galK**	Galactokinase	*zwf*	Glucose 6-phosphate-1-dehydrogenase
*galM**	Aldose 1-epimerase (mutorotase)	*focA*	Formate transport
		*ptsGHI**, *crr**	Glucose transport
*galT**	Galactose-1-phosphate uridylyltransferase	*galP**	Glucose transport (low affinity)
*galU**	UDP-glucose-1-phosphate uridylyltransferase	*glpF**	Glycerol transporter
*gapA**	Glyceraldehyde-3-phosphate dehydrogenase-A complex	*lacY**	Lactose permease
*glk**	Glucokinase	*pitAB*	Phosphate transport
*glpABC**	Glycerol-3-phosphate dehydrogenase (anaerobic)	*rbsABCD*	Ribose transport
*glpD**	Glycerol-3-phosphate dehydrogenase (aerobic)	*dctA*	Succinate transport
*glpK**	Glycerol kinase	*dcuA*	Succinate transport
gltA	Citrate synthase	*dcuB*	Succinate transport
gnd	6-Phosphogluconate dehydrogenase (decarboxylating)	*dcuC*	Succinate efflux
*gpmA**	Phosphoglycerate mutase 1	*arcA**	Aerobic/Anaerobic response regulator
*gpmB**	Phosphoglycerate mutase 2	*cra**(fruR)	Catabolite activator protein
*gpsA**	Glycerol-3-phosphate-dehydrogenase-[NAD(P)+]		
icdA	Isocitrate dehydrogenase	*dcuR*	Dicarboxylate response regulator
*lacZ**	Beta-galactosidase (LACTase)	*dcuS*	Dicarboxylate response sensor
maeB	Malic enzyme (NADP)	*fadR**	Fatty acid/Acetate response regulator
mdh	Malate dehydrogenase	*fnr**	Aerobic/Anaerobic response regulator
ndh	NADH dehydrogenase II	*galR**	Galactose operon repressor
nuoABEF− GHIJKLMN	NADH dehydrogenase I	*galS**	Galactose operon repressor
pckA	Phosphoenolpyruvate carboxykinase	*glpR**	Glycerol response regulator
*pfkA**	Phosphofructokinase	*iclR*	Fatty acid/Acetate response regulator
*pfkB**	Phosphofructokinase B	*lacI**	Lactose operon repressor
pflAB	Pyruvate formate lyase 1	*mlc**	Glucose response regulator
pflCD	Pyruvate formate lyase 2	*pdhR*	Pyruvate response regulator

Gene	Protein	Gene	Protein
*pgi**	Phosphoglucose isomerase	*rbsR*	Ribose response regulator
*pgk**	Phosphoglycerate kinase	*rpiR*	Ribose response regulator
pgl	6-Phosphogluconolactonase		

2.3 Pseudo Species

$\overline{G6P}^*$, $\overline{ACxt}$, $\overline{GLAC}^*$, $\overline{GLCxt}^*$, $\overline{GLxt}^*$, $\overline{GalR}^*$, $\overline{GalS}^*$, $\overline{GlpR}^*$, $\overline{IclR}$, $\overline{LACxt}$, $\overline{LCTSxt}^*$, $\overline{O2xt}^*$, $\overline{PYR}$, $\overline{PYRxt}$, $\overline{RIBxt}$, $\overline{SUCCxt}$, $\overline{ETHxt}$, $\overline{ArcA}^*$, $\overline{Cra}^*$, $\overline{CraCondNeg}^\dagger$, $\overline{ftktA}^\dagger$, $\overline{ftktB}^\dagger$, $\overline{ftalA}^\dagger$, $\overline{ftalB}^\dagger$, $\overline{fpgi}^\dagger$, $\overline{Fnr}^*$, $\overline{Food}^*$, $\overline{LacI}^*$, $\overline{Mlc}^*$, $\overline{PdhR}$, $\overline{PdhRCondNeg}^\dagger$, $\overline{fdld}^\dagger$, $\overline{fdctA}^\dagger$, $\overline{fdcuA}^\dagger$, $\overline{RbsR}$, $\overline{RpiR}$

2.4 Spontaneously Created Species

*ADP, ATP**, *AckA, Adk, AtpABCDEFGHI, COA, DcuA, Dld, Eno**, *FAD, FADH, Fba**, *Fbp**, *FdoIHG, GalU**, *GapA**, *Glk**, *GltA, Gnd, GpmA**, *GpmB**, *GpsA**, *HEXT**, *IcdA, MaeB, NAD**, *NADH, NADP**, *NADPH, NuoABEFGHIJKLMN, PI, PckA, PfkA**, *PfkB**, *Pgi**, *Pgl, Pgm**, *PitAB, PntAB, Ppa, Ppc, Pta, PykA**, *Q**, *QH2, Rpe, RpiA, SfcA, SucCD, TalA, TalB, TktA, TktB, TpiA**, *UTP**, *Zwf*

Species used as input species (in the complete network), respectively self-replicators (in the core network): *GLCxt*, *LCTSxt*, *GLxt*, *O2xt*

2.5 Spontaneously Decaying Species

*13PDG, 2PG, 3PG**, *AC, ACCOA, ACTP, ACxt, ADP**, *AKG, AMP**, *ATP**, *AceA, AceB, AceEF, AckA, AcnA, AcnB, Acs, AdhE, Adk, ArcA**, *AtpABCDEFGHI, BDGLAC, BDGLC, Biomass, CIT, CO2, CO2xt, COA, Cra**, *CraCondNeg, Crr**, *CydAB, CyoABCD, D6PGC, D6PGL, DctA, DcuA, DcuB, DcuC, DcuR, DcuS, Dld, E4P, ETH, ETHxt, Eno**, *F6P, FAD, FADH, FDP, FOR, FORxt, FUM, FadR**, *Fba**, *Fbp**, *FdnGHI, FdoIHG, Fnr**, *FocA, Food**, *FrdABCD, FumA, FumB, FumC, G1P, G6P**, *GAL1P**, *GL**, *GL3P, GLAC, GLC, GLCxt**, *GLX, GLxt, GalE**, *GalK**, *GalM**, *GalP**, *GalR**, *GalS**, *GalT**, *GalU**, *GapA**, *Glk**, *GlpABC**, *GlpD**, *GlpF**, *GlpK**, *GlpR**, *GltA, Gnd, GpmA**, *GpmB**, *GpsA**, *HEXT**, *ICIT, IcdA, IclR, LAC, LACxt, LCTS, LCTSxt**, *LacI**, *LacY**, *LacZ**, *LactateUP, LpdA, MAL, MaeB, Mdh, Mlc**, *NAD**, *NADH**, *NADP**, *NADPH**, *Ndh, NuoABEFGHIJKLMN, O2**, *O2xt**, *OA, PEP**, *PI**, *PIxt, PPI**, *PYR**, *PYRxt, PckA, PdhR, PdhRCondNeg, PfkA**, *PfkB**, *PflAB, PflCD, Pgi, Pgk**, *Pgl, Pgm**, *PitAB, PntAB, Ppa, Ppc, PpsA**, *Pta, PtsGHI**, *PykA**, *PykF**, *Q**, *QH2**, *R5P, RIB, RIBxt, RL5P, RbsABCD, RbsK, RbsR, Rpe, RpiA, RpiB, RpiR, S7P, SOxidRadicals, SUCC, SUCCOA, SUCCxt, SdhABCD, SfcA, SucAB, SucCD, T3P1**, *T3P2, TalA, TalB, TktA, TktB, TpiA**, *UDPG, UDPGAL, UTP**, *X5P, Zwf*

2.6 Transport Reactions

$$\begin{aligned}
HEXT+ACxt &\rightarrow AC\\
AC &\rightarrow HEXT+ACxt\\
CO2xt &\rightarrow CO2\\
CO2 &\rightarrow CO2xt\\
HEXT+ETHxt &\rightarrow ETH\\
ETH &\rightarrow HEXT+ETHxt\\
FocA+FORxt &\rightarrow FOR+FocA\\
FOR+FocA &\rightarrow FocA+FORxt\\
GLCxt+PEP+Crr+PtsGHI &\rightarrow PYR+G6P+Crr+PtsGHI+PdhRCondNeg^{\dagger *}\\
GLCxt+HEXT+GalP &\rightarrow GLC+GalP^*\\
GLxt+GlpF &\rightarrow GL+GlpF^*\\
GL+GlpF &\rightarrow GLxt+GlpF^*\\
HEXT+LACxt+LactateUP &\rightarrow LAC+LactateUP\\
LAC &\rightarrow HEXT+LACxt\\
HEXT+LCTSxt+LacY &\rightarrow LCTS+LacY^*\\
LCTS+LacY &\rightarrow HEXT+LCTSxt+LacY^*\\
O2xt &\rightarrow O2^*\\
O2 &\rightarrow O2xt^*\\
HEXT+PIxt+PitAB &\rightarrow PI+PitAB\\
PI+PitAB &\rightarrow HEXT+PIxt+PitAB\\
HEXT+PYRxt &\rightarrow PYR\\
PYR &\rightarrow HEXT+PYRxt\\
ATP+RbsABCD+RIBxt &\rightarrow ADP+PI+RIB+RbsABCD
\end{aligned}$$

$$
\begin{aligned}
\overline{FdctA}^{\dagger}+SUCCxt+HEXT+DctA &\rightarrow \overline{FdctA}^{\dagger}+PdhRCondNeg^{\dagger}+SUCC+DctA\\
FdctA^{\dagger}+SUCC+DctA &\rightarrow FdctA^{\dagger}+SUCCxt+HEXT+DctA\\
\overline{FdcuA}^{\dagger}+SUCCxt+HEXT+DcuA &\rightarrow \overline{FdcuA}^{\dagger}+PdhRCondNeg^{\dagger}+SUCC+DcuA\\
FdcuA^{\dagger}+SUCC+DcuA &\rightarrow FdcuA^{\dagger}+SUCCxt+HEXT+DcuA\\
SUCCxt+HEXT+DcuB &\rightarrow SUCC+DcuB\\
SUCC+DcuB &\rightarrow SUCCxt+HEXT+DcuB\\
SUCC+DcuC &\rightarrow SUCCxt+HEXT+DcuC
\end{aligned}
$$

2.7 Metabolic reactions

$$
\begin{aligned}
&ACCOA+PYR+PEP+T3P1+F6P+G6P\\
+OA+3PG+AKG+R5P+SUCCOA+E4P &\rightarrow Biomass\\
AceA+ICIT &\rightarrow AceA+SUCC+GLX\\
GLX+ACCOA+AceB &\rightarrow COA+AceB+MAL\\
NAD+COA+AceEF+PYR+LpdA &\rightarrow NADH+ACCOA+AceEF+LpdA+CO2\\
ADP+AckA+ACTP &\rightarrow ATP+AckA+AC\\
ATP+AckA+AC &\rightarrow ADP+AckA+ACTP\\
CIT+AcnA &\rightarrow ICIT+AcnA\\
ICIT+AcnA &\rightarrow CIT+AcnA\\
CIT+AcnB &\rightarrow ICIT+AcnB\\
ICIT+AcnB &\rightarrow CIT+AcnB\\
ATP+COA+AC+Acs &\rightarrow ACCOA+Acs+PPI+AMP\\
ACCOA+AdhE+2.0\ NADH &\rightarrow COA+AdhE+ETH+2.0\ NAD\\
COA+AdhE+ETH+2.0\ NAD &\rightarrow ACCOA+AdhE+2.0\ NADH\\
ATP+AMP+Adk &\rightarrow Adk+2.0\ ADP\\
Adk+2.0\ ADP &\rightarrow ATP+AMP+Adk\\
ATP+AtpABCDEFGHI &\rightarrow ADP+AtpABCDEFGHI+PI+4.0\ HEXT\\
ADP+AtpABCDEFGHI+PI+4.0\ HEXT &\rightarrow ATP+AtpABCDEFGHI\\
CydAB+O2+2.0\ QH2 &\rightarrow CydAB+2.0\ Q+4.0\ HEXT\\
O2+CyoABCD+2.0\ QH2 &\rightarrow CyoABCD+2.0\ Q+5.0\ HEXT\\
Fdld^{\dagger}+NADH+PYR+Dld &\rightarrow Fdld^{\dagger}+NAD+Dld+LAC\\
\overline{Fdld}^{\dagger}+NAD+Dld+LAC &\rightarrow \overline{Fdld}^{\dagger}+PdhRCondNeg^{\dagger}+NADH+PYR+Dld\\
Q+Dld+LAC &\rightarrow PdhRCondNeg^{\dagger}+QH2+PYR+Dld\\
2PG+Eno &\rightarrow Eno+PEP^{*}\\
Eno+PEP &\rightarrow 2PG+Eno^{*}\\
Fba+FDP &\rightarrow Fba+T3P2+T3P1^{*}\\
Fba+T3P2+T3P1 &\rightarrow Fba+FDP^{*}\\
FDP+Fbp &\rightarrow PI+Fbp+F6P^{*}\\
Q+FdnGHI+FOR &\rightarrow QH2+CO2+FdnGHI+2.0\ HEXT\\
Q+FOR+FdoIHG &\rightarrow QH2+CO2+FdoIHG+2.0\ HEXT\\
FADH+FrdABCD+FUM &\rightarrow FAD+SUCC+FrdABCD\\
FUM+FumA &\rightarrow MAL+FumA\\
MAL+FumA &\rightarrow FUM+FumA\\
FUM+FumB &\rightarrow MAL+FumB\\
MAL+FumB &\rightarrow FUM+FumB\\
FUM+FumC &\rightarrow MAL+FumC\\
MAL+FumC &\rightarrow FUM+FumC\\
GalE+UDPGAL &\rightarrow GalE+UDPG^{*}\\
GalE+UDPG &\rightarrow GalE+UDPGAL^{*}\\
ATP+GLAC+GalK &\rightarrow ADP+GalK+GAL1P^{*}\\
ADP+GalK+GAL1P &\rightarrow ATP+GLAC+GalK^{*}\\
BDGLAC+GalM &\rightarrow GLAC+GalM^{*}\\
GLAC+GalM &\rightarrow BDGLAC+GalM^{*}\\
GalM+BDGLC &\rightarrow GalM+GLC^{*}\\
GalM+GLC &\rightarrow GalM+BDGLC^{*}\\
UTP+GAL1P+GalT &\rightarrow PPI+UDPGAL+GalT^{*}\\
PPI+UDPGAL+GalT &\rightarrow UTP+GAL1P+GalT^{*}
\end{aligned}
$$

$UTP+GalU+G1P \rightarrow PPI+UDPG+GalU^*$

$PPI+UDPG+GalU \rightarrow UTP+GalU+G1P^*$

$NAD+PI+T3P1+GapA \rightarrow NADH+GapA+13PDG^*$

$NADH+GapA+13PDG \rightarrow NAD+PI+T3P1+GapA^*$

$ATP+GLC+Glk \rightarrow ADP+Glk+G6P^*$

$Q+GlpABC+GL3P \rightarrow QH2+T3P2+GlpABC^*$

$Q+GL3P+GlpD \rightarrow QH2+T3P2+GlpD^*$

$ATP+GL+GlpK \rightarrow ADP+GL3P+GlpK^*$

$ACCOA+OA+GltA \rightarrow COA+CIT+GltA$

$NADP+Gnd+D6PGC \rightarrow NADPH+CO2+Gnd+RL5P$

$3PG+GpmA \rightarrow 2PG+GpmA^*$

$2PG+GpmA \rightarrow 3PG+GpmA^*$

$3PG+GpmB \rightarrow 2PG+GpmB^*$

$2PG+GpmB \rightarrow 3PG+GpmB^*$

$NADP+GL3P+GpsA \rightarrow NADPH+T3P2+GpsA^*$

$NADPH+T3P2+GpsA \rightarrow NADP+GL3P+GpsA^*$

$NADP+ICIT+IcdA \rightarrow NADPH+CO2+IcdA+AKG$

$NADPH+CO2+IcdA+AKG \rightarrow NADP+ICIT+IcdA$

$LCTS+LacZ \rightarrow bDGLAC+GLC+LacZ^*$

$NADP+MAL+MaeB \rightarrow NADPH+PYR+CO2+MaeB$

$NAD+MAL+Mdh \rightarrow NADH+OA+Mdh$

$NADH+OA+Mdh \rightarrow NAD+MAL+Mdh$

$NADH+Q+Ndh \rightarrow NAD+QH2+Ndh$

$NuoABEFGHIJKLMN+2.0\ NADH+2.0\ Q \rightarrow NuoABEFGHIJKLMN+2.0\ NAD+2.0\ QH2+7.0\ HEXT$

$ATP+OA+PckA \rightarrow ADP+CO2+PEP+PckA$

$ATP+F6P+PfkA \rightarrow ADP+FDP+PfkA^*$

$ATP+F6P+PfkB \rightarrow ADP+FDP+PfkB^*$

$COA+PYR+PflAB \rightarrow ACCOA+FOR+PflAB$

$COA+PYR+PflCD \rightarrow ACCOA+FOR+PflCD$

$\overline{fpgi}^{\dagger}+G6P+Pgi \rightarrow \overline{fpgi}^{\dagger}+CraCondNeg^{\dagger}+F6P+Pgi^*$

$fpgi^{\dagger}+F6P+Pgi \rightarrow fpgi^{\dagger}+G6P+Pgi^*$

$ADP+13PDG+Pgk \rightarrow ATP+3PG+Pgk^*$

$ATP+3PG+Pgk \rightarrow ADP+13PDG+Pgk^*$

$Pgl+D6PGL \rightarrow D6PGC+Pgl$

$G1P+Pgm \rightarrow G6P+Pgm^*$

$G6P+Pgm \rightarrow G1P+Pgm^*$

$NAD+NADPH+PntAB \rightarrow NADH+NADP+PntAB$

$NADH+NADP+PntAB+2.0\ HEXT \rightarrow NAD+NADPH+PntAB$

$PPI+Ppa \rightarrow Ppa+2.0\ PI$

$CO2+PEP+Ppc \rightarrow PI+OA+Ppc$

$ATP+PYR+PpsA \rightarrow AMP+PI+PEP+PpsA^*$

$ACCOA+PI+Pta \rightarrow COA+ACTP+Pta$

$COA+ACTP+Pta \rightarrow ACCOA+PI+Pta$

$ADP+PEP+PykA \rightarrow ATP+PYR+PykA+PdhRCondNeg^{\dagger *}$

$ADP+PEP+PykF \rightarrow ATP+PYR+PykF+PdhRCondNeg^{\dagger *}$

$ATP+RIB+RbsK \rightarrow ADP+RbsK+R5P$

$RL5P+Rpe \rightarrow Rpe+X5P$

$Rpe+X5P \rightarrow RL5P+Rpe$

$RL5P+RpiA \rightarrow R5P+RpiA$

$R5P+RpiA \rightarrow RL5P+RpiA$

$RL5P+RpiB \rightarrow R5P+RpiB$

$R5P+RpiB \rightarrow RL5P+RpiB$

$FAD+SUCC+SdhABCD \rightarrow FADH+FUM+SdhABCD$

$Q+FADH+SdhABCD \rightarrow QH2+FAD+SdhABCD$

$QH2+FAD+SdhABCD \rightarrow Q+FADH+SdhABCD$

$NAD+MAL+SfcA \rightarrow NADH+PYR+CO2+SfcA$

$NAD+COA+LpdA+AKG+SucAB \rightarrow NADH+CO2+SUCCOA+LpdA+SucAB$

$ADP+PI+SUCCOA+SucCD \rightarrow ATP+COA+SUCC+SucCD$

$ATP+COA+SUCC+SucCD \rightarrow ADP+PI+SUCCOA+SucCD$

$$
\begin{aligned}
\overline{ftalA}^{\dagger}+T3P1+S7P+TalA &\rightarrow \overline{ftalA}^{\dagger}+CraCondNeg^{\dagger}+F6P+TalA+E4P\\
ftalA^{\dagger}+F6P+TalA+E4P &\rightarrow ftalA^{\dagger}+T3P1+S7P+TalA\\
\overline{ftalB}^{\dagger}+T3P1+S7P+TalB &\rightarrow \overline{ftalB}^{\dagger}+CraCondNeg^{\dagger}+F6P+E4P+TalB\\
ftalB^{\dagger}+F6P+E4P+TalB &\rightarrow ftalB^{\dagger}+T3P1+S7P+TalB\\
R5P+X5P+TktA &\rightarrow T3P1+S7P+TktA\\
T3P1+S7P+TktA &\rightarrow R5P+X5P+TktA\\
\overline{ftktA}^{\dagger}+X5P+E4P+TktA &\rightarrow \overline{ftktA}^{\dagger}+CraCondNeg^{\dagger}+T3P1+F6P+TktA\\
ftktA^{\dagger}+T3P1+F6P+TktA &\rightarrow ftktA^{\dagger}+X5P+E4P+TktA\\
R5P+X5P+TktB &\rightarrow T3P1+S7P+TktB\\
T3P1+S7P+TktB &\rightarrow R5P+X5P+TktB\\
\overline{ftktB}^{\dagger}+X5P+E4P+TktB &\rightarrow \overline{ftktB}^{\dagger}+CraCondNeg^{\dagger}+T3P1+F6P+TktB\\
ftktB^{\dagger}+T3P1+F6P+TktB &\rightarrow ftktB^{\dagger}+X5P+E4P+TktB\\
T3P1+TpiA &\rightarrow T3P2+TpiA^{*}\\
T3P2+TpiA &\rightarrow T3P1+TpiA^{*}\\
NADP+G6P+Zwf &\rightarrow NADPH+D6PGL+Zwf\\
NADPH+D6PGL+Zwf &\rightarrow NADP+G6P+Zwf\\
ATP &\rightarrow ADP+PI^{*}
\end{aligned}
$$

2.8 Regulatory Reactions

$$
\begin{aligned}
\overline{IclR} &\rightarrow \overline{IclR}+AceA\\
\overline{IclR}+\overline{ArcA} &\rightarrow \overline{IclR}+\overline{ArcA}+AceB\\
\overline{PdhR} &\rightarrow \overline{PdhR}+AceEF+LpdA\\
Food &\rightarrow AcnA+Food\\
Food &\rightarrow AcnB+Food\\
\overline{IclR}+\overline{GLCxt}+\overline{LCTSxt}+\overline{RIBxt}&\\
+\overline{GLxt}+\overline{LACxt}+\overline{PYRxt}+\overline{SUCCxt}+\overline{ETHxt} &\rightarrow \overline{IclR}+\overline{GLCxt}+\overline{LCTSxt}+\overline{RIBxt}\\
&\qquad +\overline{GLxt}+\overline{LACxt}+\overline{PYRxt}+\overline{SUCCxt}+\\
&\qquad \overline{ETHxt}+Acs\\
\overline{O2xt} &\rightarrow \overline{O2xt}+AdhE\\
\overline{Fnr} &\rightarrow \overline{Fnr}+CydAB\\
ArcA &\rightarrow CydAB+ArcA\\
\overline{ArcA}+\overline{Fnr} &\rightarrow \overline{ArcA}+\overline{Fnr}+CyoABCD\\
Fnr &\rightarrow FdnGHI+Fnr\\
Fnr &\rightarrow FrdABCD+Fnr\\
FDP+Fbp+\overline{craCondNeg}^{\dagger} &\rightarrow FDP+Fbp+\overline{craCondNeg}^{\dagger}+Cra\\
DcuR &\rightarrow FrdABCD+DcuR\\
\overline{ArcA}+\overline{Fnr} &\rightarrow \overline{ArcA}+\overline{Fnr}+FumA\\
Fnr &\rightarrow FumB+Fnr\\
SOxidRadicals &\rightarrow FumC+SOxidRadicals\\
\overline{GLCxt}+\overline{GalR}+\overline{GalS} &\rightarrow \overline{GLCxt}+\overline{GalR}+\overline{GalS}+GalE^{*}\\
\overline{GLCxt}+\overline{GalR}+\overline{GalS} &\rightarrow \overline{GLCxt}+\overline{GalR}+\overline{GalS}+GalK^{*}\\
\overline{GLCxt}+\overline{GalR}+\overline{GalS} &\rightarrow \overline{GLCxt}+\overline{GalR}+\overline{GalS}+GalM^{*}\\
\overline{GLCxt}+\overline{GalR}+\overline{GalS} &\rightarrow \overline{GLCxt}+\overline{GalR}+\overline{GalS}+GalT^{*}\\
\overline{GLCxt}+\overline{GlpR}+\overline{LCTSxt}+\overline{RIBxt}+Fnr &\rightarrow \overline{GLCxt}+\overline{GlpR}+\overline{LCTSxt}+\overline{RIBxt}+\\
&\qquad GlpABC+Fnr^{*1}\\
\overline{GLCxt}+\overline{GlpR}+\overline{LCTSxt}+\overline{RIBxt}+\overline{ArcA} &\rightarrow \overline{GLCxt}+\overline{GlpR}+\overline{LCTSxt}+\overline{RIBxt}+\overline{ArcA}+\\
&\qquad GlpD^{*1}\\
\overline{GLCxt}+\overline{GlpR}+\overline{LCTSxt}+\overline{RIBxt} &\rightarrow \overline{GLCxt}+\overline{GlpR}+\overline{LCTSxt}+\overline{RIBxt}+GlpK^{*1}\\
\overline{GLCxt}+\overline{LacI} &\rightarrow \overline{GLCxt}+\overline{LacI}+LacZ^{*}\\
\overline{ArcA} &\rightarrow \overline{ArcA}+Mdh\\
\overline{Fnr} &\rightarrow \overline{Fnr}+Ndh\\
ArcA &\rightarrow PflAB+ArcA\\
Fnr &\rightarrow PflAB+Fnr\\
ArcA &\rightarrow PflCD+ArcA\\
Fnr &\rightarrow PflCD+Fnr\\
Food &\rightarrow Pgk+Food^{*}\\
Cra &\rightarrow PpsA+Cra^{*}\\
\overline{Cra} &\rightarrow \overline{Cra}+PykF^{*}
\end{aligned}
$$

$$
\begin{aligned}
MAL+MaeB+\overline{PdhRCondNeg}^{\dagger} &\rightarrow MAL+MaeB+\overline{PdhRCondNeg}^{\dagger}+PdhR \\
MAL+SfcA+\overline{PdhRCondNeg}^{\dagger} &\rightarrow MAL+SfcA+\overline{PdhRCondNeg}^{\dagger}+PdhR \\
\overline{GLCxt}+\overline{LCTSxt}+\overline{RbsR} &\rightarrow \overline{GLCxt}+\overline{LCTSxt}+\overline{RbsR}+RbsK \\
\overline{RpiR} &\rightarrow \overline{RpiR}+RpiB \\
\overline{ArcA}+\overline{Fnr} &\rightarrow \overline{ArcA}+\overline{Fnr}+SdhABCD \\
\overline{PdhR} &\rightarrow \overline{PdhR}+LpdA+SucAB \\
ArcA &\rightarrow FocA+ArcA \\
Fnr &\rightarrow FocA+Fnr \\
\overline{Mlc}+Food &\rightarrow \overline{Mlc}+Crr+PtsGHI+Food^{*} \\
\overline{Cra}+Food &\rightarrow \overline{Cra}+Crr+PtsGHI+Food^{*} \\
Food &\rightarrow GalP+Food^{*} \\
\overline{GLCxt}+\overline{GlpR}+\overline{LCTSxt}+\overline{RIBxt} &\rightarrow \overline{GLCxt}+\overline{GlpR}+\overline{LCTSxt}+\overline{RIBxt}+GlpF^{*\,1} \\
\overline{GLCxt}+\overline{GLxt}+\overline{LCTSxt}+\overline{RIBxt} &\rightarrow \overline{GLCxt}+\overline{GLxt}+\overline{LCTSxt}+\overline{RIBxt}+ \\
&\qquad LactateUP \\
\overline{GLCxt}+\overline{LacI} &\rightarrow \overline{GLCxt}+\overline{LacI}+LacY^{*} \\
\overline{GLCxt}+\overline{LCTSxt}+\overline{RbsR} &\rightarrow \overline{GLCxt}+\overline{LCTSxt}+\overline{RbsR}+RbsABCD \\
\overline{GLCxt}+\overline{GLxt}+\overline{LACxt}+\overline{LCTSxt}+ & \\
\overline{PYRxt}+\overline{RIBxt}+\overline{ArcA}+DcuR &\rightarrow \overline{GLCxt}+\overline{GLxt}+\overline{LACxt}+\overline{LCTSxt}\ + \\
&\qquad +\ \overline{PYRxt}+\overline{RIBxt}+\overline{ArcA}+DctA+DcuR \\
\overline{GLCxt}+\overline{GLxt}+\overline{LACxt}+\overline{LCTSxt}+ & \\
\overline{PYRxt}+\overline{RIBxt}+Fnr+DcuR &\rightarrow \overline{GLCxt}+\overline{GLxt}+\overline{LACxt}+\overline{LCTSxt} \\
&\qquad +\ \overline{PYRxt}+\overline{RIBxt}+DcuB+Fnr+DcuR \\
\overline{ArcA}+\overline{Fnr} &\rightarrow \overline{ArcA}+\overline{Fnr}+DcuC \\
\overline{O2xt} &\rightarrow \overline{O2xt}+ArcA^{*} \\
\overline{G6P}+FDP+fbp &\rightarrow \overline{G6P}+FDP+fbp+cra \\
DcuS &\rightarrow DcuR+DcuS \\
SUCCxt &\rightarrow SUCCxt+DcuS \\
GLCxt &\rightarrow GLCxt+FadR^{*} \\
\overline{ACxt} &\rightarrow \overline{ACxt}+FadR \\
\overline{O2xt} &\rightarrow \overline{O2xt}+Fnr^{*} \\
\overline{LCTSxt} &\rightarrow \overline{LCTSxt}+GalR^{*} \\
\overline{LCTSxt} &\rightarrow \overline{LCTSxt}+GalS^{*} \\
\overline{GLxt} &\rightarrow \overline{GLxt}+GlpR^{*} \\
FadR &\rightarrow FadR+IclR \\
\overline{LCTSxt} &\rightarrow \overline{LCTSxt}+LacI^{*} \\
\overline{GLCxt} &\rightarrow \overline{GLCxt}+Mlc^{*} \\
\overline{PYR} &\rightarrow \overline{PYR}+pdhR \\
\overline{RIBxt} &\rightarrow \overline{RIBxt}+RbsR \\
\overline{RIBxt} &\rightarrow \overline{RIBxt}+RpiR \\
GLCxt &\rightarrow GLCxt+Food^{*} \\
LCTSxt &\rightarrow LCTSxt+Food^{*} \\
RIBxt &\rightarrow RIBxt+Food \\
GLxt &\rightarrow GLxt+Food^{*} \\
LACxt &\rightarrow LACxt+Food \\
PYRxt &\rightarrow PYRxt+Food \\
SUCCxt &\rightarrow SUCCxt+Food \\
ETHxt &\rightarrow ETHxt+Food \\
ACxt &\rightarrow ACxt+Food \\
FORxt &\rightarrow FORxt+Food
\end{aligned}
$$

In reactions marked [1], $\overline{RIBxt}$ was removed from the reaction in the core network model.

3 Analysis of a Genome-Scale Metabolic Model

In order to present cases in which OT leads to more accurate predictions compared to FBA, we take a genome-scale metabolic model by Reed et al. [2]. In order to consider cellular growth, we add a decay reaction for each metabolite. The growth medium is represented by a set of influx reactions according to Table 7.

Table 7: Growth medium for the model of Reed et al. [2].

**Growth medium**
CO_2, Fe^{2+}, H+, water, potassium, natrium, ammonium, anorganic phosphate, sulfate, oxygen, D-glucose, acetate, glycerol, D-lactate, succinate

3.1 Role of Co-factors

One reason for wrong predictions by FBA is that FBA takes co-factors insufficiently into account. Co-factors are molecules that are necessary for some reactions to proceed. They can interact through various means with the substrates and products of a reaction.

Some of the reactions producing metabolites appearing in the biomass reaction can only proceed if certain co-factors are present. These can be molecules which participate in a reaction but are released and reconverted to their initial form in a later step. Most of these co-factors are included in the biomass reaction, but some of them are not. This can for example be the case, if these co-factors are in reality proteins or RNAs which have just been included if an intermediate metabolite is covalently bound to them.

3.1.1 Problem of Non-Metabolic Species

Whether FBA classifies a knockout as viable is not changed by adding a decay for each metabolite. However, OT reveals that this "prediction" is wrong, because in the model by Reed et al. under growth-conditions, there is no organization containing biomass. Consequently, there is no long-term behavior at which biomass is produced and thus there cannot be growth.

An analysis of this problem revealed, that this model includes metabolites that account for tRNA, the acyl carrier protein (Acp), and thioredoxin. Acp and thioredoxin participate in reactions that are essential for the production of biomass metabolites. They are only used as "co-factors" for these reactions. However, there are no production pathways for them.

The addition of an inflow for each of these metabolites then yields a biomass producing organization. We take now the resulting model in order to predict knock experiments.

As can be expected, the knockout of these production pathways is correctly predicted as lethal by OT. FBA however does neither take into account the decay, nor the production reaction, and hence predicts that the organism is viable, independently of whether the production reaction is present or not.

3.1.2 Problem of Redundant Co-factors

Another case arises, when co-factors can be substituted by other co-factors, which is for example the case for menaquinone and ubiquinone in *E. coli* (Figure 1). There is also a third metabolite, 2-demethylmenaquinone, which can be used if both, menaquinone and ubiquinone, cannot be produced by the cell [3]. However, the model we investigated cannot produce biomass when the fluxes of all reactions in which menaquinone and ubiquinone appear are constrained to zero (neither in FBA nor in OT). Thus, here, we only consider menaquinone and ubiquinone.

The presence of either species is sufficient to produce biomass (Figure 1). Thus, including them both in the biomass producing reaction would yield false results when considering knockouts in pathway for the production of one of them. This might be the reason, why both metabolites have not been included in the biomass reaction. The synthesis of both metabolites uses chorismate and octaprenyl as intermediate metabolites, which is also necessary for the production of 2-demethylmenaquinone. The knockouts of each gene essential in the octaprenyl biosynthesis is predicted as lethal by OT, while FBA predicts a viable organism. *In vivo* each of this knockout is lethal [4]. The corresponding genes are listed in Table 8

Another case arises in the knockout of *uppS*, which is correctly predicted as lethal by OT, but not by FBA. This gene synthesizes undecaprenyl diphosphate, a metabolite which acts as co-factor in the lipid metabolism. A more recent model of the metabolism of *E. coli* by Feist et al. [5] includes this metabolite in the biomass function, while neither menaquinone nor ubiquinone are included.

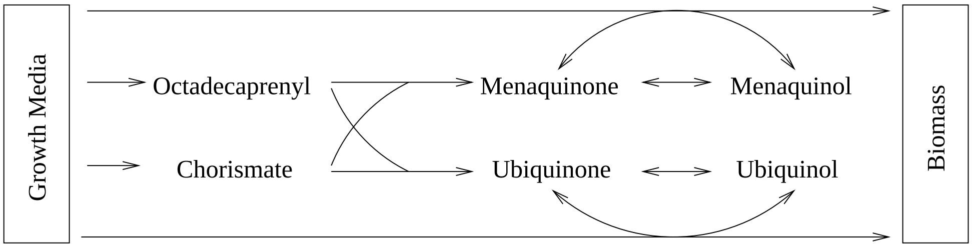

Figure 1: Schematic drawing of the role of menaquinone and ubiquinone in the production of biomass metabolites. The presence of either metabolite gives rise to an organization producing biomass. Knockouts in the essential reactions of the pathway leading to the production of octadecaprenyl are correctly predicted as lethal by OT and falsely predicted as viable by FBA. Please note that both the oxidized and the reduced versions of menaquinone and ubiquinone can be involved in the synthesis of biomass metabolites, thus the corresponding arrows are drawn in both directions.

Table 8: List of 10 lethal knockouts [4] that are correctly predicted by OT, in contrast to FBA.

**Pathway**	**Essential genes**
menaquinone, ubiquinone, 2-demethylmenaquinone production	*dxr, dxs, gcpE, ispA, ispB, ispD, ispE, ispF, lytB*
undecaprenyl diphosphate production	*uppS*

4 A Flux Based Algorithm for Computing Organizations

We briefly review our algorithm that computes all organizations of a given reaction network (a detailed presentation and analysis will be published elsewhere). For large networks, the computation of all organizations does not finish in reasonable time. We describe a heuristic approach that can at least compute a subset of all organizations in this case.

4.1 Using the self-maintenance cone in flux-space

To be an organization, a set of species has to fulfill two properties: closure and self-maintenance. Starting with the latter condition, methods from convex analysis can be employed to compute organizations. Given a reaction network $\langle \mathcal{M}, \mathcal{R} \rangle$ and its $m \times n$ stoichiometric matrix $\mathbf{S}$, a flux vector $v \in \mathbb{R}^n$ fulfilling the self-maintenance condition must be found to show that a species set is self-maintaining. All such flux vectors lie in a convex polyhedral cone $\mathcal{P}$ in the n-dimensional flux space $\mathbb{R}^n$, originating in the point of origin. The cone is defined by the $n + m$ inequalities:

$$v \geq \mathbf{0} \text{ and } \mathbf{S} \cdot v \geq \mathbf{0}.$$

The constraints can be transformed into a matrix $\mathcal{A}$ representing the extreme rays or spanning vectors of $\mathcal{P}$ [6]. Each point within $\mathcal{P}$ can be written as a linear combination of these extreme rays. Thus, we can compute organizations by searching for combinations of extreme rays whose corresponding set of species fulfills the closure condition.

To compute the extreme rays for a given cone $\mathcal{P}$, we implemented the well-known Schuster algorithm [7] to compute elementary modes. This algorithm computes the extreme rays of a convex cone $\mathcal{P}'$ defined by $v \geq \mathbf{0}$ and $\mathbf{S} \cdot v = \mathbf{0}$. By adding an outflux for each metabolite to the stoichiometric matrix used for the elementary mode computation, the algorithm can also compute the extreme rays of the cone $\mathcal{P}$.

4.2 The extreme ray algorithm

The extreme ray algorithm takes as input a reaction network $\langle \mathcal{M}, \mathcal{R} \rangle$ and a set of extreme rays V_B defining the convex polyhedral cone $\mathcal{P}$ containing all self-maintenance flux vectors. The algorithm delivers all organizations of the network. If instead a convex polyhedral cone $\mathcal{P}' \subset \mathcal{P}$ is supplied as input, only those organizations are computed, whose self-maintaining flux vectors lie within $\mathcal{P}'$. This is useful when, for example, only balanced organizations are to be computed. The cone $\mathcal{P}'$ containing all steady state flux distribution then serves as input for the algorithm. Given a flux vector v, the algorithm only relies on the set of reactions that have positive fluxes in v and not on the specific flux values. Hence, we define v^{set} as the set of reaction indices containing all reactions that have positive fluxes in v. Considering the set of extreme rays V_B defining $\mathcal{P}$, V_B^{set} describes the set of reaction sets v_B^{set} corresponding to the extreme

rays $v_B \in V_B$. Note that generally $|V^{set}| \leq |V|$, as a reaction set v^{set} can correspond to more than one flux vector v. The species set that corresponds to a reaction set v^{set} is denoted by $M(v^{set})$. It contains all reactants and products of the reactions contained in v^{set}.

The computation of the organizations is split into three parts. In the first part, all elementary organizations are computed. An organization O is elementary if no other organizations exist such that the union of their reactions equals the reactions of O. The elementary organizations are then used in the second part of the algorithm to find all organizations that have different sets of reactions. In the final step, organizations are computed that have the same set of reactions like the already computed organizations, but contain different sets of species. In this step organizations are discovered that contain isolated species that do not participate in any reaction of the organization.

4.2.1 Step 1: computing elementary organizations

The central function in the computation of elementary organizations is *organizationsAbove()*. Given a self-maintenance flux vector $v \in \mathcal{P}$ and its corresponding reaction set v^{set}, it computes all organizations O that contain $M(v^{set})$ and for which there exists no other organization being a subset of O and a superset of $M(v^{set})$. (More precisely, *at least* those organizations are computed. Under certain circumstances, organizations are also in the result set for which a subset O_s is also an organization and contains $M(v^{set})$. However, in such a case O_s is also contained in the resulting set of organizations.) Hence, the smallest organizations containing $M(v^{set})$ are computed.

First, the closure of the reaction set v^{set}, respectively $M(v^{set})$, is computed. This is done by taking the species set $M(v^{set})$ and iteratively adding all species to the set that can be created by reactions of the network from the species set. The reaction set $v^{set}_{Closure}$ contains all reactions that can take place in the generated closed set of species. If this reaction set is identical to v^{set}, the species set $M(v^{set})$ is an organization. The reaction set $v^{set}_{Closure}$ contains more reactions than v^{set} when either species were added, or $M(v^{set})$ is closed but v^{set} does not contain all reactions that are possible in this set. One such reaction is taken, and all reaction sets $v^{set}_B \in V^{set}_B$ that contain this reaction are consecutively combined with the original reaction set v^{set} and the function is called again recursively. As the initial reaction set v^{set} and the extreme ray reaction sets v^{set}_B correspond to flux vectors fulfilling the self-maintenance condition, also a flux vector v_u fulfilling the self-maintenance property exists for the union $v^{set}_u = v^{set} \cup v^{set}_B$. Hence, all reaction sets that are considered in the recursive function calls are associated with self-maintaining flux vectors. To obtain all elementary organizations, the function *organizationsAbove()* is called for each reaction set $v^{set}_B \in V^{set}_B$ corresponding to an extreme ray defining $\mathcal{P}$. The central function as pseudo code:

Function `organizationsAbove`

Input: reaction network $\langle \mathcal{M}, \mathcal{R} \rangle$, set V^{set}_B of reaction sets corresponding to the extreme rays spanning the self-maintenance cone $\mathcal{P}$, reaction set v^{set} corresponding to a self-maintenance flux vector $v \in \mathcal{P}$

Output: the set of the smallest organizations containing the reactions of v^{set}

result $\leftarrow \{\}$;
$v^{set}_{Closure} \leftarrow$ `closure(`v^{set} `)`;
if $v^{set}_{Closure} == v^{set}$ **then**
  result $\leftarrow \{$ `M(`v^{set} `)`$\}$;
else
  select one reaction r with $r \in (v^{set}_{Closure} \setminus v^{set})$;
  foreach $v^{set}_B \in V^{set}_B$ *with* $r \in v^{set}_B$ **do**
    result = result $\cup$ `organizationsAbove(`$v^{set} \cup v^{set}_B$ `)`;
  end
end
`return(`*result* `)` ;

4.2.2 Step 2 & 3: computing all organizations

In the first step of the algorithm, the function *organizationsAbove()* was called for each of the reaction sets corresponding to the extreme rays defining $\mathcal{P}$.

In the second part, organizations are determined that are combinations of elementary organizations. This is done by taking all possible combinations of two elementary organizations and calling *organizationsAbove()* for the union of their reaction sets. For every newly discovered organization, this organization must be again combined with each of the elementary organizations and *organizationsAbove()* must be called again for the reaction set unions.

The organizations we have obtained so far all possess a different set of reactions. Consequently, the third step consists of searching for organizations having the same set of reactions as already discovered ones. Hence, we need to determine for all discovered organizations all species sets, that can be added to the organization without changing its set of reactions.

4.3 An heuristic approach to organization computation

The presented algorithm requires the extreme rays of the cone $\mathcal{P}$ as input. For larger networks, the time needed for their computation exceeds practical limits. In order to compute at least a subset of all organizations in such cases, a heuristic approach can be employed. Instead of starting with the set of all extreme rays V_B, the first step of the algorithm can be skipped by directly starting with a set of elementary organizations. To obtain such a set, a simple heuristic approach is used. A random walk through the reaction network delivers a set of species. After computing the closure of this species set, it is tested whether the closure also fulfills the self-maintenance condition. This is done by solving the linear programming problem defined by the self-maintenance constraints and a dummy objective function. After having determined a sufficient large set of organizations, the associated set of elementary organizations O_{el} is determined. The reaction sets of the organizations in O_{el} are then used as input to the second step of the extreme ray algorithm to compute the complete set of organizations that can be found by combining organizations from O_{el}. The heuristic approach was able to correctly determine the whole set of organizations for all tested networks to which we could also apply the exact method.

To verify the results, each knockout experiment has been run two times independently for two hours. Than it was checked whether the predictions for both runs were equal. Since we found no differences, we consider the results reliable.

References

[1] M. W. Covert and B. Palsson. Transcriptional regulation in constraints-based metabolic models of escherichia coli. *J Biol Chem*, 277(31):28058–28064, Aug 2002.

[2] Jennifer L Reed, Thuy D Vo, Christophe H Schilling, and Bernhard O Palsson. An expanded genome-scale model of escherichia coli k-12 (ijr904 gsm/gpr). *Genome Biol*, 4(9):R54, 2003. doi: 10.1186/gb-2003-4-9-r54. URL `http://dx.doi.org/10.1186/gb-2003-4-9-r54`.

[3] P. T. Lee, A. Y. Hsu, H. T. Ha, and C. F. Clarke. A c-methyltransferase involved in both ubiquinone and menaquinone biosynthesis: isolation and identification of the escherichia coli ubie gene. *J Bacteriol*, 179(5):1748–1754, Mar 1997.

[4] Tomoya Baba, Takeshi Ara, Miki Hasegawa, Yuki Takai, Yoshiko Okumura, Miki Baba, Kirill A Datsenko, Masaru Tomita, Barry L Wanner, and Hirotada Mori. Construction of escherichia coli k-12 in-frame, single-gene knockout mutants: the keio collection. *Mol Syst Biol*, 2:2006.0008, 2006. doi: 10.1038/msb4100050. URL `http://dx.doi.org/10.1038/msb4100050`.

[5] Adam M Feist, Christopher S Henry, Jennifer L Reed, Markus Krummenacker, Andrew R Joyce, Peter D Karp, Linda J Broadbelt, Vassily Hatzimanikatis, and Bernhard Palsson. A genome-scale metabolic reconstruction for escherichia coli k-12 mg1655 that accounts for 1260 orfs and thermodynamic information. *Mol Syst Biol*, 3:121, 2007. doi: 10.1038/msb4100155. URL `http://dx.doi.org/10.1038/msb4100155`.

[6] Julien Gagneur and Steffen Klamt. Computation of elementary modes: a unifying framework an the new binary approach. *BMC Bioinformatics*, 5:175, 2004.

[7] S. Schuster, T. Dandekar, and D. A. Fell. Detection of elementary flux modes in biochemical networks: a promising tool for pathway analysis and metabolic engineering. *Trends Biotechnol*, 17(2):53–60, Feb 1999.
